# Knockout of a *PLD* gene in *Schizochytrium limacinum* SR21 enhances docosahexaenoic acid accumulation by modulation of the phospholipid profile

**DOI:** 10.1186/s13068-024-02465-w

**Published:** 2024-01-30

**Authors:** Yiting Zhang, Xiaowen Cui, Shuizhi Lin, Tao Lu, Hao Li, Yinghua Lu, Mingfeng Cao, Xihuang Lin, Xueping Ling

**Affiliations:** 1 Department of Chemical and Biochemical Engineering, College of Chemistry and Chemical Engineering, Xiamen University, Xiamen, People’s Republic of China; 2 Xiamen Key Laboratory of Synthetic Biotechnology, Xiamen University, Xiamen, People’s Republic of China; 3 The Key Laboratory for Chemical Biology of Fujian Province (Xiamen University), Xiamen, People’s Republic of China; 4 Analysis and Test Center, Ministry of Natural Resources, Third Institute of Oceanography, Xiamen, 361005 People’s Republic of China

**Keywords:** *Schizochytrium limacinum*, Phospholipase D, Docosahexaenoic acid, Phospholipomics, Acyl CoA-independent pathway

## Abstract

**Background:**

The hydrolysis and transphosphatidylation of phospholipase D (PLD) play important roles in the interconversion of phospholipids (PLs), which has been shown to profoundly impact lipid metabolism in plants. In this study, the effect of the *PLD1* gene of *Schizochytrium limacinum* SR21 (*S. limacinum* SR21) on lipid metabolism was investigated.

**Results:**

*PLD1* knockout had little impact on cell growth and lipid production, but it significantly improved the percentage of polyunsaturated fatty acids in lipids, of which docosahexaenoic acid (DHA) content increased by 13.3% compared to the wild-type strain. Phospholipomics and real-time quantitative PCR analysis revealed the knockout of *PLD1* reduced the interexchange and increased de novo synthesis of PLs, which altered the composition of PLs, accompanied by a final decrease in phosphatidylcholine (PC) and an increase in phosphatidylinositol, lysophosphatidylcholine, and phosphatidic acid levels. *PLD1* knockout also increased DHA content in triglycerides (TAGs) and decreased it in PLs.

**Conclusions:**

These results indicate that *PLD1* mainly performs the transphosphatidylation activity in *S. limacinum* SR21, and its knockout promotes the migration of DHA from PLs to TAGs, which is conducive to DHA accumulation and storage in TAGs via an acyl CoA-independent pathway. This study provides a novel approach for identifying the mechanism of DHA accumulation and metabolic regulation strategies for DHA production in *S. limacinum* SR21.

**Supplementary Information:**

The online version contains supplementary material available at 10.1186/s13068-024-02465-w.

## Background

*Schizochytrium* is a natural representative strain for the industrial production of docosahexaenoic acid (DHA) owing to its fast growth rate and high lipid content, containing more than 50% DHA [1–4]. DHA is a very important unsaturated fatty acid for the human body, which can promote the growth and maintenance of nervous system cells. It is an essential component in the retina and brain. Therefore, this substance has a great promoting effect on the development of infants' vision and intelligence. It can also improve memory and reduce the occurrence of postpartum depression in pregnant women. At present, the DHA yield of *Schizochytrium limacinum* is basically maintained at 6.52–14 g/L [5, 6] by shake flask culture. The mechanism of DHA synthesis in *Schizochytrium* has been extensively studied and is known to involve two distinct biochemical pathways: the aerobic fatty acid synthesis (FAS) pathway and the anaerobic polyketide synthase (PKS) pathway [7–12], which are the main targets for regulating the synthesis of polyunsaturated fatty acids (PUFAs) in microorganisms [13–16]. Recent studies have revealed that DHA production depends not only on the pathway of DHA synthesis but also on the migration, accumulation, and assembly of DHA stored after synthesis [17, 18]. Although DHA is mainly stored as DHA-triglyceride (DHA-TAG) in *Schizochytrium* and *Thraustochytrium* [19], it has been reported that the synthesized DHA might first be incorporated into phospholipids (PLs) to form DHA-PLs, before being channeled and assembled into DHA-TAG for storage [20, 21]. This suggests that the final DHA yield is closely related to PL metabolism. PLs are the main components of cell membranes and play important roles in biological reproduction, cell division, and membrane transport [22]. The regulation of PL metabolism in *Schizochytrium* may be effective for DHA production.

Phospholipase D (PLD) is capable of converting different types of PLs through transphosphatidylation or hydrolyzing the polar head of PLs to produce phosphatidic acid (PA) [23], which has a significant role in cell growth and lipid metabolism [24, 25]. Currently, studies on PLD expression in the regulation of lipid metabolism mainly focus on plants [26, 27]. Overexpression of the soybean (*Glycine max*) *PLDγ* gene (*GmPLDγ*) in *Arabidopsis* resulted in increased seed weight, a greater number of smaller lipid droplets, and significant upregulation of glycerolipid metabolism-related genes, suggesting a regulatory role for *GmPLDγ* in TAG synthesis and fatty acid remodelling [28]. Knockout of *PLDα1* in soybean (*Glycine max*) resulted in the higher unsaturation of TAG and major membrane lipids and lower unsaturation of PA and lysophospholipids during seed development. The study also found that phospholipid:diacylglycerol acyltransferase (PDAT) facilitated the conversion of acyl-CoA between phosphatidylcholine (PC) and diacylglycerol (DAG), promoting the unsaturation of TAG [29]. These results suggest that the regulation of *PLD* expression exerts an important influence on lipid metabolism in plants, particularly on the allocation of fatty acids between TAG and PLs. There are few publications on the function of *PLD* in lipid synthesis of microorganisms; therefore, it is of interest to explore the effect of *PLD* regulation on the patterns of PLs and DHA production in *Schizochytrium limacinum* SR21 (*S. limacinum* SR21) [30], a premium engineered strain used for lipid production.

In this study, we first mined two *PLD* genes, *PLD1* and *PLD2*, from *S. limacinum* SR21 and constructed the corresponding knockout strains, ∆*PLD1* and ∆*PLD2*. The mutant strain with the higher DHA yield, ∆*PLD1*, was further analyzed using phospholipomics and real-time quantitative PCR (qRT-PCR) to reveal the potential regulatory mechanism of *PLD* on DHA production in *S. limacinum* SR21.

## Results and discussion

### Mining of *PLD* genes

**Fig. 1:**
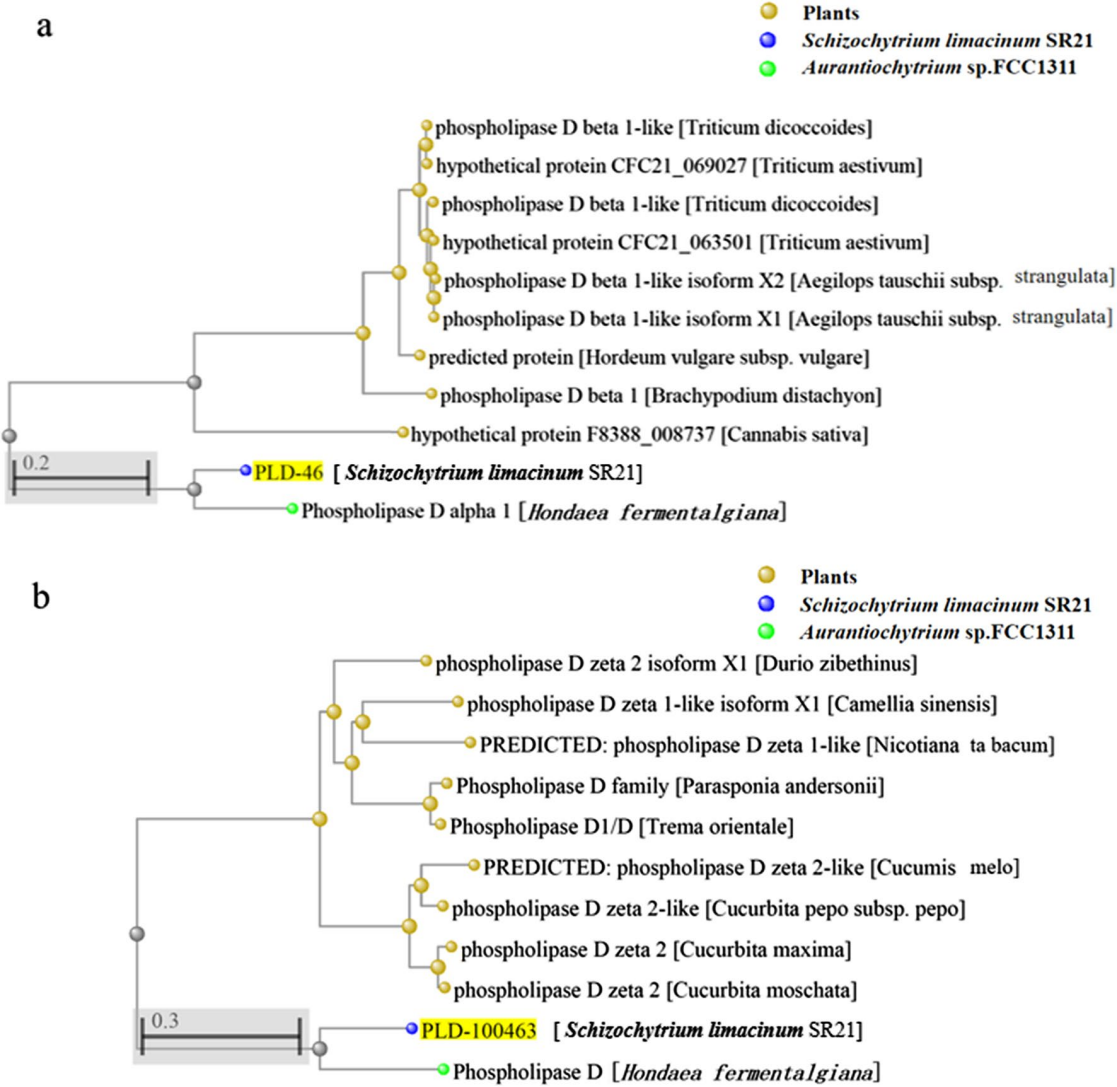
Phylogenetic tree analysis of the (**a**) *PLD-46* and (**b**) *PLD-100463* gene sequences of *S. limacinum* SR21. Bootstrap values (> 50%, repeated 1,000 times) are displayed on each internal branch

Seventeen species of PLD corresponding to the EC3.1.4.4 enzyme classification were found in the JGI database. The sequence similarity of the *PLD* genes and the corresponding proteins among species was not high, indicating that the PLDs found in the JGI database were independent and non-repetitive (Additional file 1: Fig. S1). Notably, PLD superfamily domains were found in PLD-46 and PLD-100463 proteins. Phylogenetic tree analysis of *PLD-46* and *PLD-100463* genes showed that they were closely related to the *PLD* and *PLDα1* genes of *Aurantiochytrium* sp. FCC1311, respectively, and had 59.20 and 67.02% sequence homology, respectively (Fig. 1). Therefore, in this study, we chose to regulate the expression of *PLD-46* and *PLD-100463*, which were named *PLD1* and *PLD2*, respectively*.* Gene sequences are listed in Additional file 1: Figs. S2, S3.

### Construction of transgenic *S. limacinum* SR21 with knockout of *PLD1 *or* PLD2*

Both positive transformants were screened for zeocin resistance. The PCR validations of the zeocin gene are shown in Additional file 1: Fig. S4. The target band of the zeocin resistance gene (375 bp) appeared in the knockout strains and positive control groups but not in the negative control group, indicating that the *PLD1* or *PLD2* gene had been successfully knocked out in *S. limacinum* SR21.

### Effects of* PLD1 *or* PLD2* knockout on cell growth and lipid synthesis

**Fig. 2:**
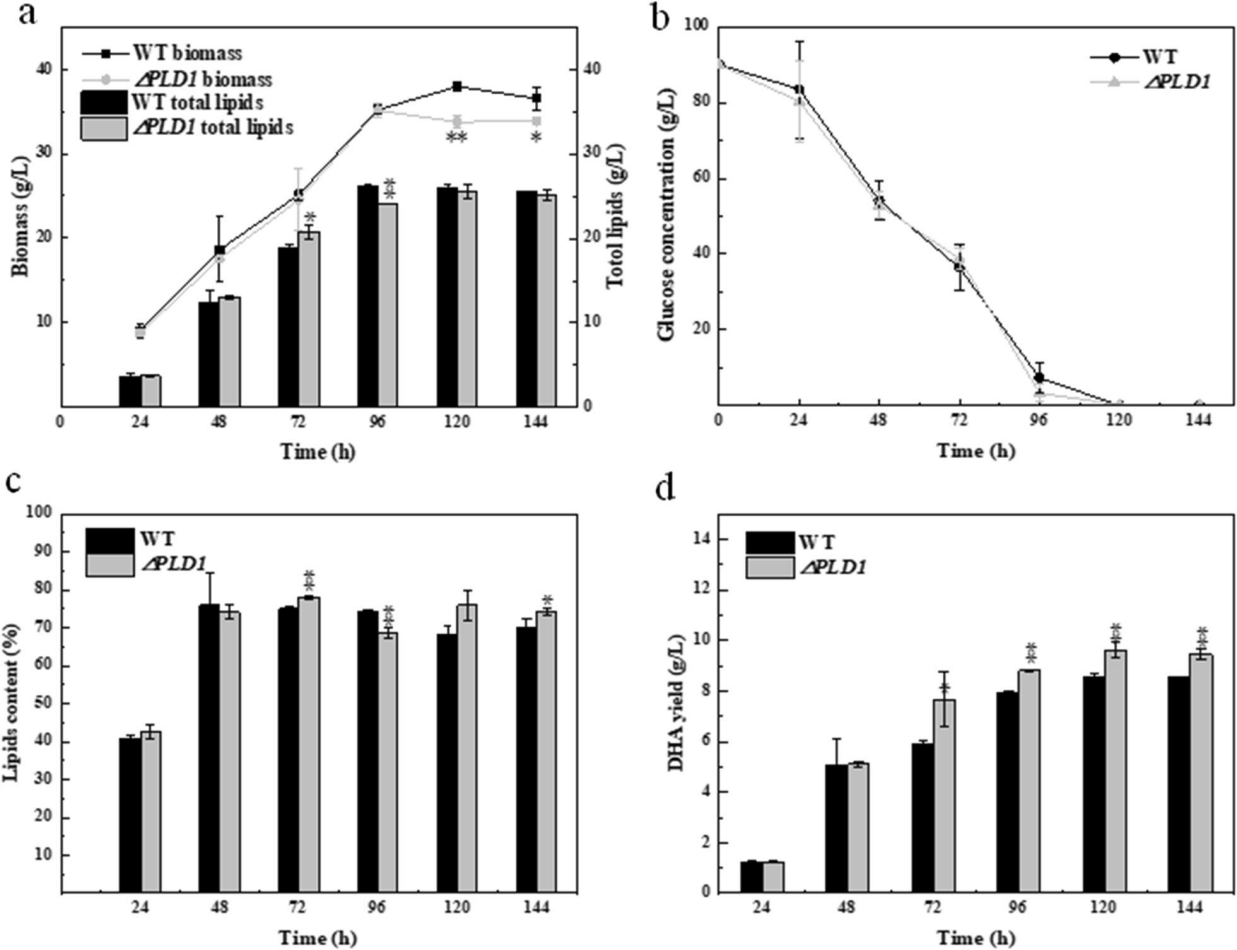
(**a**) Biomass and total lipids, (**b**) glucose concentration, (**c**) lipid content, and (**d**) DHA yield of the wild-type and the Δ*PLD1* strains. * and ** represent 0.01 < *p* < 0.05 and *p* < 0.01, respectively. All data are expressed as mean ± SD of three independent experiments

As shown in Fig. 2a, compared with the wild type, there was no significant change in the biomass of the ∆*PLD1* strain before 96 h, but a decrease of 11.0% (*p* < 0.01) and 7.4% (*p* < 0.05) occurred at 120 and 144 h, respectively. During the whole fermentation, the total lipid yield of ∆*PLD1* strain showed little change when compared with the wild type. Figure 2b shows that the ∆*PLD1* and wild-type strains had the same glucose consumption rate throughout the fermentation process. Glucose was almost completely used up at 96 h, which was consistent with the observations of the same cell growth patterns in both strains before 96 h. The small decrease in biomass in the ∆*PLD1* strain at the later stage was probably the result of knocking out *PLD1.* The lipid content in the two strains also showed no significant change during the full growth stage (Fig. 2c); however, DHA production was improved in the ∆*PLD1* strain (Fig. 2d). The highest yield of DHA from shake flask fermentation of ∆*PLD1* strain achieved 9.61 g/L, which was 12.3% higher than that of the wild type (*p* < 0.01). These results suggest that knockout of *PLD1* may slightly affect cell membrane function, resulting in minor cell damage, which does not impact total lipid production but enhances DHA accumulation.

**Fig. 3:**
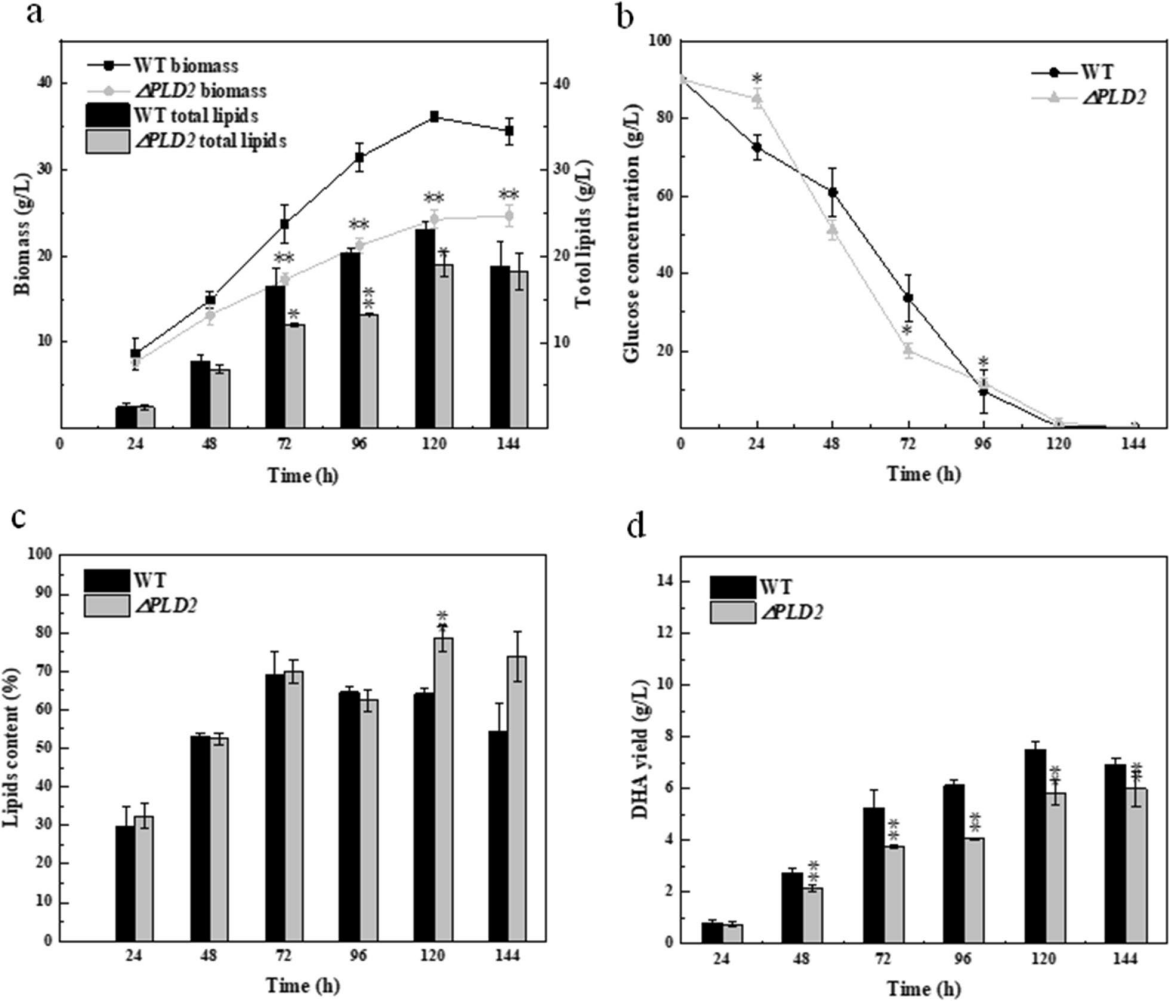
(**a**) Biomass and total lipids, (**b**) glucose concentration, (**c**) lipid content, and (**d**) DHA yield of the wild-type and the Δ*PLD2* strains. * and ** represent 0.01 < *p* < 0.05 and *p* < 0.01, respectively. All data are expressed as mean ± SD of three independent experiments

As shown in Fig. 3a, compared with the wild type, the biomass of the **∆***PLD2* strain declined drastically from 72 to 144 h, and the highest decrease was observed at 120 h (32.8%; *p* < 0.01). The total lipids of the **∆***PLD2* strain showed a similar reduction as that observed for biomass, decreasing by 17.6% (*p* < 0.01) at 120 h. Figure 3b shows that the glucose consumption rate in the **∆***PLD2* strain decreased at the early stage but increased at the middle stage compared to the wild-type strain. The **∆***PLD2* strain consumed the same amount of glucose as the wild-type strain. The lipid content of the **∆***PLD2* strain showed no apparent change compared to that of the wild type before 96 h (Fig. 3c) but was higher at 120 and 144 h. This indicated that the two strains had the same ability to produce lipids before 96 h, and that the **∆***PLD2* strain could improve the ability of the unit cell to produce lipids after 120 h. We postulated that the knockout of *PLD2* severely impaired cell membrane function, leading to decreased cell growth and lipid yield. To maintain a certain amount of growth, cells of **∆***PLD2* strain used glucose to synthesize other metabolites that resist cell damage and after 120 h, mature cells of the **∆***PLD2* strain showed enhanced lipid synthesis. DHA production in the *PLD2* knockout strain decreased significantly during all stages compared to the wild-type strain (Fig. 3d). These results indicated that *PLD2* knockout is not conducive to cell growth, total lipid production, or DHA yield.

### Effect of *PLD1* or *PLD2* knockout on fatty acid composition

Table 1 shows that the *PLD1* knockout decreased the percentage of saturated fatty acids (SFAs) and increased the proportion of PUFAs in the lipids of *Schizochytrium*. In the ∆*PLD1* strain, the proportion of DHA in lipids increased by 13.3% and 12.5% at 120 and 144 h, respectively. These results suggest that *PLD1* knockout facilitates the synthesis of PUFAs, particularly DHA, in *Schizochytrium*. Knockdown of the *PLDα1* gene in soybeans resulted in higher unsaturation of TAG [29], which is similar to the results of the present study.

**Table 1:** Fatty acid composition of wild-type strain and ∆*PLD1* strain at late stages of fermentation

Fatty acid	120 h			144 h		
	WT	∆*PLD1*	Significance	WT	∆*PLD1*	Significance
C14:0	3.38 ± 0.03	3.78 ± 0.02	**	3.20 ± 0.07	3.78 ± 0.08	**
C15:0	1.27 ± 0.03	0.96 ± 0.01	**	1.24 ± 0.04	0.94 ± 0.01	**
C16:0	49.76 ± 0.20	43.20 ± 0.20	**	49.05 ± 0.34	42.96 ± 0.99	**
C17:0	0.48 ± 0.01	0.30 ± 0.00	**	0.49 ± 0.02	0.30 ± 0.00	**
C18:0	1.74 ± 0.01	1.67 ± 0.02	**	1.85 ± 0.03	1.63 ± 0.01	**
EPA	0.39 ± 0.03	0.36 ± 0.00		0.48 ± 0.01	0.39 ± 0.01	**
DPA	6.92 ± 0.03	8.32 ± 0.01	**	7.10 ± 0.09	8.38 ± 0.13	*
DHA	33.11 ± 0.16	37.52 ± 0.18	**	33.52 ± 0.31	37.69 ± 0.80	**
Others	2.94 ± 0.12	3.89 ± 0.11	*	3.09 ± 0.09	3.94 ± 0.18	**
SFAs	56.63 ± 0.20	49.91 ± 0.23	**	55.81 ± 0.48	49.60 ± 1.10	**
PUFAs	40.43 ± 0.16	46.19 ± 0.19	**	41.10 ± 0.41	46.46 ± 0.92	**

When *p* > 0.05, the difference is insignificant; when 0.01 < *p* < 0.05, the difference is significant and represented by *; when *p* < 0.01, the difference is extremely significant and represented by **

Table 2 shows that the *PLD2* knockout had little effect on the proportion of PUFAs and SFAs in *Schizochytrium*. However, a decrease in the proportion of even-numbered carbon fatty acids, such as C14:0, C16:0, and C18:0, was observed, and the proportion of odd-numbered carbon fatty acids (OCFAs) increased significantly, of which C15:0 and C17:0 increased by 246.2% (*p* < 0.01) and 259.6% (*p* < 0.01), respectively. OCFA concentration in human plasma has been reported to be negatively correlated with the risk of type 2 diabetes and cardiovascular disease [16, 31, 32]; therefore, increasing the production of OCFAs helps increase the commercial potential of *Schizochytrium*. The above results suggest that *PLD2* knockout mainly inhibited cell growth to reduce lipid yield but did not affect the synthesis pathway of PUFAs and SFAs in *Schizochytrium*.

**Table 2:** Fatty acid composition of wild-type strain and △*PLD2* strain at late stages of fermentation

Fatty acid	120 h			144 h		
	WT	△*PLD2*	Significance	WT	△*PLD2*	Significance
C14:0	2.96 ± 0.17	2.08 ± 0.14	**	2.83 ± 0.46	1.92 ± 0.07	**
C15:0	1.22 ± 0.30	4.00 ± 0.32	**	1.00 ± 0.05	3.47 ± 0.52	**
C16:0	50.77 ± 0.57	48.81 ± 1.13	**	50.51 ± 0.55	46.54 ± 0.75	**
C17:0	0.56 ± 0.16	2.29 ± 0.25	**	0.46 ± 0.06	2.09 ± 0.27	**
C18:0	1.65 ± 0.03	1.88 ± 0.06	**	1.74 ± 0.12	1.89 ± 0.03	**
EPA	0.42 ± 0.05	0.35 ± 0.02		0.57 ± 0.14	0.55 ± 0.12	**
DPA	6.90 ± 0.35	6.64 ± 0.21	**	6.95 ± 0.17	7.06 ± 0.38	*
DHA	32.48 ± 0.69	30.63 ± 0.26	**	32.34 ± 0.35	32.80 ± 0.68	**
Others	3.04 ± 0.13	3.32 ± 0.39	*	3.60 ± 0.86	3.68 ± 0.37	**
SFAs	57.15 ± 1.06	59.06 ± 0.68	**	56.54 ± 0.79	55.91 ± 1.53	**
PUFAs	39.80 ± 1.09	37.62 ± 0.48	**	39.86 ± 0.28	40.41 ± 1.18	**
OCFAs	1.78 ± 0.46	6.29 ± 0.57	**	1.46 ± 0.10	5.56 ± 0.78	**

When *p* > 0.05, the difference is insignificant; when 0.01 < *p* < 0.05, the difference is significant and represented by *; when *P* < 0.01, the difference is extremely significant and represented by **

The regulation of *PLD* can influence phospholipid metabolism by playing hydrolysis and transphosphatidylation, and subsequently affect the function of the cell membrane and the glycerophospholipid metabolic pathway, thereby influencing the cell growth and lipid metabolism of *Schizochytrium* [33, 34]. The different results for the *PLD1* and *PLD2* knockouts may be related to their preference for hydrolysis or transphosphatidylation, allowing them to have different roles in phospholipid metabolism. PLD1 is thought to play a role in transphosphatidylation, which affects phospholipid composition and the regulation of fatty acid synthesis, whereas *PLD2* mainly hydrolyzes phospholipid, the knockout of which damages cell function and inhibits cell growth and lipid production. The functional relationship between them deserves to be further explored. Given that the *PLD1* knockout promoted DHA synthesis without a significant effect on cell growth and lipid production, whereas *PLD2* knockout showed a severe reduction in cell growth and lipid synthesis compared to the wild strain, the ∆*PLD1* strain was chosen for the subsequent experiments and analysis.

### Effect of *PLD1* knockout on transcriptional levels of related genes

**Fig. 4:**
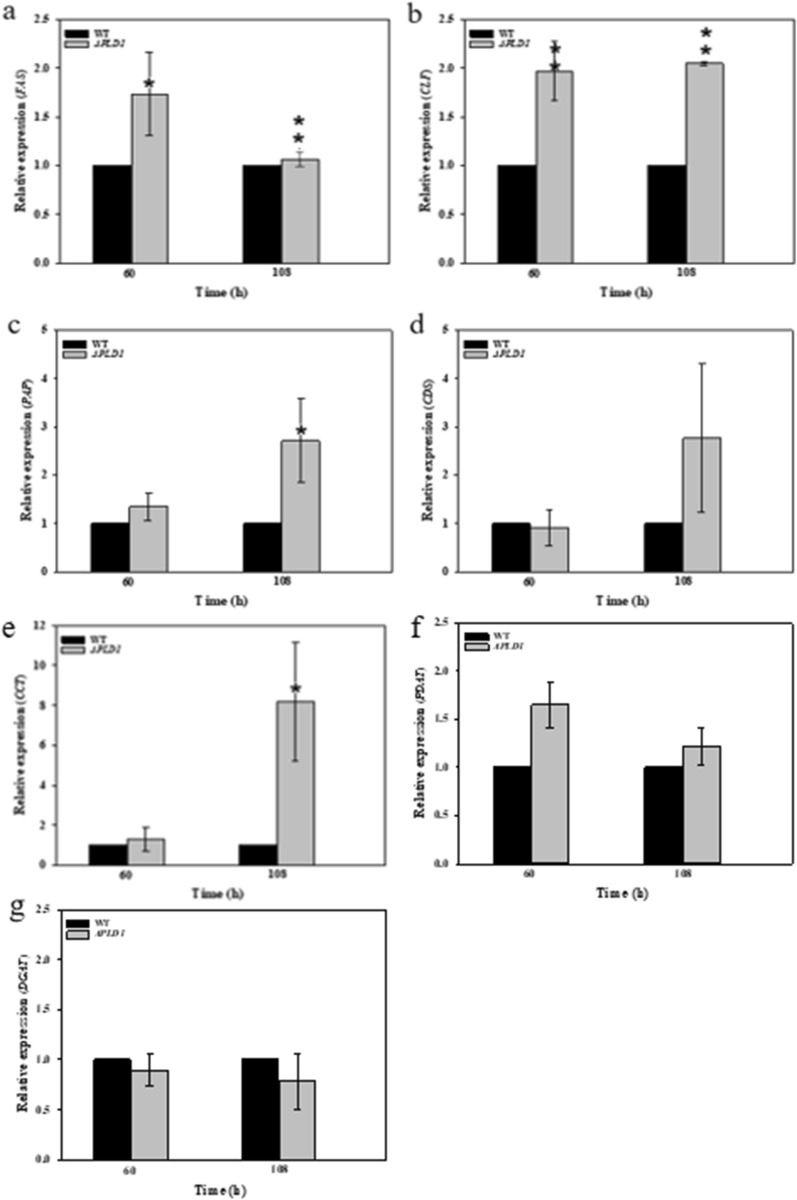
Transcription levels of related genes in wild-type and Δ*PLD1* strains: (**a**) fatty acid synthase (*FAS*), (**b**) chain length factor (*CLF*), (**c**) phospholipid acid phosphatase (*PAP*), (**d**) CDP-diacylglycerol synthase (*CDS*), (**e**) choline phosphate transferase (*CCT*), (**f**) phosphatidylglycerol acyltransferase (*PDAT*), (**g**) diacylglycerol acyltransferase (*DGAT*). There was a significant difference at the 0.01 < *p* < 0.05 (*) or *p* < 0.01 (**) level between the wild-type strain and the Δ*PLD1* strain. All data were expressed as mean ± SD of three independent experiments

The transcriptional levels of genes involved in lipid metabolism in the wild-type and knockout strains were measured during the logarithmic growth period (60 h) and lipid transformation period (108 h) (Fig. 4). The fatty acid synthase (*FAS*) gene encodes a series of proteins related to the de novo synthesis of fatty acids in the form of gene clusters that can synthesize saturated acetyl CoA via the traditional FAS pathway [35]. The chain length factor (*CLF*) gene is located in the PKS gene cluster and is responsible for chain lengthening during PUFA synthesis [36]. At 60 h, expression levels of both *FAS* and *CLF* genes were significantly upregulated in the ∆*PLD1* strain; at 108 h, in the lipid transformation stage, the expression of *CLF* in the ∆*PLD1* strain increased by 96.6%, whereas the expression of *FAS* did not change significantly (Fig. 4a, b). This supports the observation that *PLD1* knockout enhanced DHA synthesis (Fig. 2d). Phosphatidic acid phosphatase (PAP) catalyzes the synthesis of DAG from PA, a hydrolytic product of PLD. *PLD1* knockout improved the expression level of *PAP*, which increased by 1.71 times in the lipid transformation stage compared to the wild-type strain (Fig. 4c). This results in more PA being transformed into DAG in the ∆*PLD1* strain, suggesting that PLD1 may play a greater role in transphosphatidylation than hydrolysis. *PLD1* knockout regulates phospholipid metabolism by weakening the transphosphatidylation of phospholipids, thereby promoting the Kennedy pathway and improving TAG synthesis. CDP-diacylglycerol synthase (CDS) and phosphocholine cytidyl transferase (CCT) are the key enzymes involved in phospholipid synthesis, including that of phosphatidylserine (PS), phosphatidylinositol (PI), phosphatidylglycerol (PG), and PC. At the 108 h timepoint, the relative transcription level of *CDS* and *CCT* in the ∆*PLD1* were 1.76 and 7.18 times higher than those in the wild-type strain (Fig. 4d, e), respectively. This indicates that *PLD1* knockout increases the synthesis of phospholipids and further demonstrates that PLD1 is not responsible for hydrolysis of phospholipids in *Schizochytrium*. If PLD1 played a role in hydrolytic activity, the ∆*PLD1* strain would accumulate the hydrolytic substrates of phospholipids and the hydrolytic product of PA would be reduced, thereby inhibiting the synthetic pathway of phospholipids and TAG synthesis from PA. Therefore, it is assumed that PLD1 regulates phospholipid composition by the transphosphatidylation of phospholipids, which influences fatty acid synthesis and DHA accumulation in *Schizochytrium*.

For TAG synthesis, PDAT uses PC as an acyl donor and DAG as an acyl acceptor to transfer the acyl group of a phospholipid to DAG [29]. PLD1 knockout promoted PDAT transcription at 60 and 108 h (Fig. 4f). Correspondingly, the transcriptional level of the diacylglycerol acyltransferase gene (*DGAT*), which catalyzes DAG and acyl-CoA to form TAG via the Kennedy pathway, was downregulated at both stages (Fig. 4g). These results indicate that the knockout of *PLD1* increases TAG synthesis via the acyl-CoA-independent pathway of PDAT and reduces TAG synthesis via the Kennedy pathway of DGAT, which favors the production of unsaturated TAG [29]; this promotes DHA accumulation in TAG.

### Effects of *PLD1* knockout on phospholipid metabolism

#### Phospholipid content and fatty acid composition

**Fig. 5:**
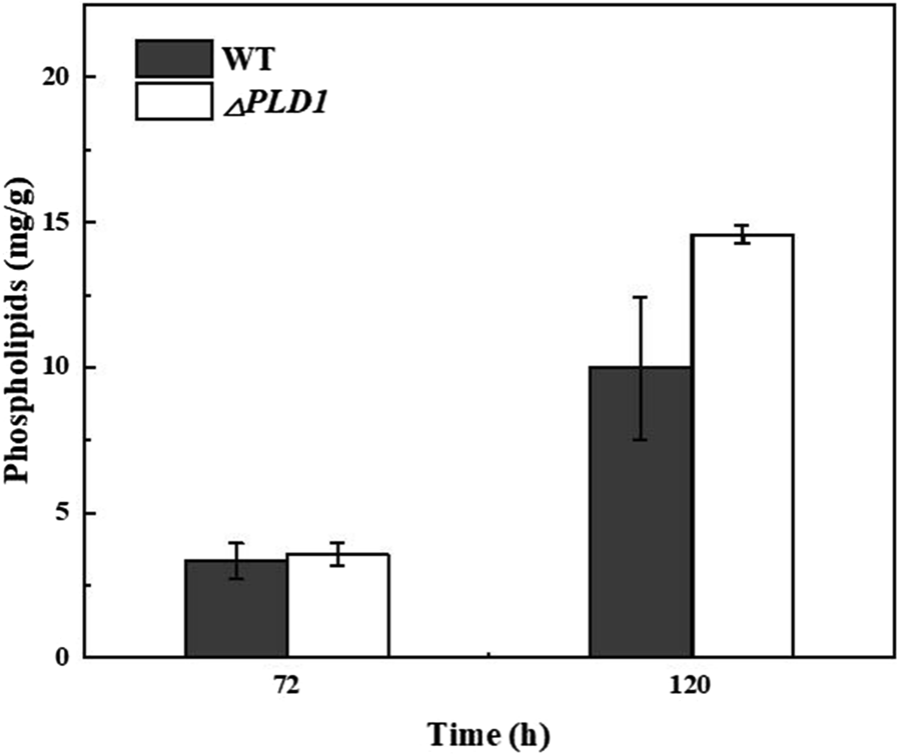
Phospholipid content in 72 and 120 h samples of the wild-type and Δ*PLD*1 strains. All data were expressed as mean ± standard deviation (mean ± SD) of three independent experiments

As shown in Fig. 5, *PLD1* knockout led to an apparent increase in total PLs at 120 h, probably because *PLD1* knockout reduced the transesterification activity of PLD, resulting in weakened interconversion and enhanced de novo synthesis of PLs (Fig. 4d, e). In *Thraustochytrium* sp. 26185, DHA preferentially integrates into PLs rather than directly into TAG and then migrates to TAG to form DHA-TAGs at a later stage [10]. Yue [20] et al. confirmed that DHA accumulates in both TAG and PC during the growth of *Schizochytrium* sp. A-2 and that DHA migrated from PC to TAG at a later stage of fermentation. The increase in total phospholipids may facilitate the assembly and accumulation of DHA, which could also explain the increased DHA production caused by *PLD1* knockout (Fig. 2d).

Table 3 shows the main bound fatty acids in the TAGs and PLs of *S. limacinum* SR21. Both TAG and PLs mainly bound SFAs, which accounted for approximately 40–55%, of which C16:0 accounted for the largest proportion (> 90%). However, the proportions of PUFAs in the TAG and PLs were significantly different. The proportion of PUFAs in TAGs was approximately 35%, including DHA, DPA, and EPA, whereas PUFAs in PLs only accounted for 10–15%, mainly consisting of DHA and DPA. These results indicate that PUFAs or (DHA) are mainly bound to TAG. Notably, EPA was not detected in the PLs and was thought to be at too low a level to be detected. This suggests that EPA was mostly bound to TAG, which provides a new theory for the study of EPA synthesis in *S. limacinum* SR21. Comparison of the fatty acid composition at different time points showed that the content of PUFAs or DHA in the TAG of both strains was higher at 120 h than at 72 h, and the content of SFAs decreased accordingly. However, the content of PUFAs or DHA in PLs at 120 h was lower than that at 72 h. It was assumed that DHA migrated from PLs to TAG in the later stages, which is consistent with previously reported results [18, 19]. Comparison of the fatty acid composition of the different strains at 72 and 120 h showed that the *PLD1* knockout increased the content of PUFAs and DHA in TAG and decreased the content of DHA in PLs, indicating that *PLD1* knockout promoted the unsaturation of TAGs. Zhang et al. found the knockout of *PLDα1* in soybean (*Glycine max*) resulted in the higher unsaturation of TAG, which was catalyzed by PDAT to shift the unsaturated acyl-CoA from PC to TAG [29]. PDAT-mediated TAG synthesis pathway was also reported to be conducive to the channeling of DHA from PC to TAG in the later fermentation stage in *Schizochytrium* [20]. The improved expression of *CCT* (Fig. 4e) and PDAT (Fig. 4f) in the ∆*PLD1* strain suggests the enhanced PC synthesis and the increased conversion of acyl-CoA from PC to TAG. Combined with the content change of DHA in TAGs and PLs, it’s inferred that the knockout of *PLD1* promotes the migration of DHA from PLs to TAG, which finally enhances the accumulation and storage of DHA (Fig. 2d).

**Table 3:** Main fatty acid composition in TAG and PLs of wild-type strains and *△PLD1* strains

Culture condition	Lipid class	C14:0	Percent of fatty acids (%)						PUFAs
			C16:0	C18:0	SFAs	EPA	DPA	DHA	
WT 72 h	TAG	0.12 ± 0.04	52.0 ± 3.53	2.43 ± 0.71	54.6 ± 4.26	0.14 ± 0.03	5.64 ± 0.82	28.8 ± 0.5	34.6 ± 0.59
	PL	2.03 ± 0.9	34.8 ± 13.57	13.7 ± 2.55	50.5 ± 15.1	0.00 ± 0.00	3.18 ± 0.44	11.1 ± 2.07	14.3 ± 1.63
*△PLD1* 72 h	TAG	0.17 ± 0.03	44.0 ± 4.75	2.57 ± 0.41	46.7 ± 4.72	0.18 ± 0.03	7.71 ± 0.63	32.6 ± 4.88	40.5 ± 5.52
	PL	2.55 ± 0.35	28.9 ± 1.04	17.0 ± 0.83	48.5 ± 1.38	0.00 ± 0.00	1.05 ± 0.44	3.81 ± 0.23	4.86 ± 0.67
WT 120 h	TAG	0.30 ± 0.09	46.9 ± 2.04	2.86 ± 0.32	50.1 ± 2.45	0.22 ± 0.06	6.02 ± 0.80	30.7 ± 2.27	36.9 ± 1.53
	PL	1.26 ± 0.16	25.7 ± 12.91	13.2 ± 0.49	40.2 ± 12.6	0.00 ± 0.00	3.09 ± 1.23	6.69 ± 0.72	9.78 ± 0.94
*△PLD1* 120 h	TAG	0.26 ± 0.21	42.6 ± 1.01	1.67 ± 0.51	44.5 ± 1.17	0.20 ± 0.08	7.75 ± 1.04	34.0 ± 1.70	42.0 ± 0.62
	PL	2.30 ± 0.83	20.9 ± 2.99	11.2 ± 2.15	34.4 ± 5.77	0.00 ± 0.00	0.57 ± 0.21	3.22 ± 0.75	3.79 ± 0.72

#### Phospholipid types and composition in *S. limacinum* SR21

The polar head structure of phospholipids determines the phospholipid species. As shown in Table 4, 43 phospholipid molecules were identified; including 15 PC, nine lysophosphatidylcholine (LPC), 10 PG, four phosphatidylethanolamine (PE), two PI, two PS, and one PA. The three main phospholipids in *S. limacinum* SR21 were PC, PI, and PG; and PC accounted for half of the total PLs. PC is also the main phospholipid in other *Schizochytrium* strains [20, 37, 38] and is related to DHA accumulation and migration to TAG [20, 21]. PI has an inositol head group that plays an important role in cell signal transduction and metabolic regulation [39]. PG is closely related to cell growth and affects the lipid dependence of cellular stress responses and adaptation mechanisms [40]. The levels of PE, PS, and PA in *S. limacinum* SR21 were low (< 5%); however, they also play important roles in cell membrane structure and function [33].

**Table 4:** Phospholipid species in *S. limacinum* SR21 (120 h)

Type	Unsaturation	Acyl composition	Molecular formula	m/z	Content (μg/g)
PC	C30:0	C14:0/C16:0	C_38_H_76_O_8_NP	750.5285	6.45
	C32:0	C16:0/C16:0	C_40_H_80_O_8_NP	778.5598	16.10
	C36:5	C14:0/C22:5	C_44_H_78_O_8_NP	824.5442	15.53
	C36:6	C14:0/C22:6	C_44_H_76_O_8_NP	822.5285	18.19
	C37:5	C15:0/C22:5	C_45_H_80_O_8_NP	838.5598	6.45
	C37:6	C15:0/C22:6	C_45_H_78_O_8_NP	836.5442	19.19
	C38:5	C16:0/C22:5	C_46_H_82_O_8_NP	852.5755	661.44
	C38:6	C16:0/C22:6	C_46_H_80_O_8_NP	850.5598	2076.51
	C40:6	C18:0/C22:6	C_48_H_84_O_8_NP	878.5911	14.32
	C40:7	C18:1/C22:6	C_48_H_82_O_8_NP	876.5755	28.22
	C42:10	C20:4/C22:6	C_50_H_80_O_8_NP	898.5598	124.96
	C42:11	C20:5/C22:6	C_50_H_78_O_8_NP	896.5442	270.73
	C44:10	C22:5/C22:5	C_52_H_84_O_8_NP	926.5911	58.27
	C44:11	C22:5/C22:6	C_52_H_82_O_8_NP	924.5755	525.61
	C44:12	C22:6/C22:6	C_52_H_80_O_8_NP	922.5598	1518.21
LPC	C14:0	C14:0	C_22_H_46_NO_7_P	512.2988	4.19
	C15:0	C15:0	C_23_H_48_NO_7_P	526.3145	1.30
	C16:0	C16:0	C_24_H_50_NO_7_P	540.3301	59.88
	C18:0	C18:0	C_26_H_54_NO_7_P	568.3614	1.10
	C18:1	C18:1	C_26_H_52_NO_7_P	566.3458	2.79
	C18:2	C18:2	C_26_H_50_NO_7_P	564.3301	2.28
	C20:5	C20:5	C_28_H_48_NO_7_P	586.3145	2.44
	C22:5	C22:5	C_30_H_52_NO_7_P	614.3458	109.23
	C22:6	C22:6	C_30_H_50_NO_7_P	612.3301	489.56
PG	C30:0	C14:0/C16:0	C_36_H_71_O_10_P	693.4707	0.35
	C31:0	C15:0/C16:0	C_37_H_73_O_10_P	707.4863	1.20
	C32:0	C16:0/C16:0	C_38_H_75_O_10_P	721.502	147.72
	C38:5	C16:0/C22:5	C_44_H_77_O_10_P	795.5176	11.97
	C38:6	C16:0/C22:6	C_44_H_75_O_10_P	793.502	98.12
	C40:6	C18:0/C22:6	C_46_H_79_O_10_P	821.5333	0.80
	C40:7	C18:1/C22:6	C_46_H_77_O_10_P	819.5176	1.34
	C42:10	C20:4/C22:6	C_48_H_75_O_10_P	841.502	3.60
	C44:11	C22:5/C22:6	C_50_H_77_O_10_P	867.5176	2.57
	C44:12	C22:6/C22:6	C_50_H_75_O_10_P	865.502	5.03
PI	C38:5	C16:0/C22:5	C_47_H_81_O_13_P	883.5337	1069.19
	C38:6	C16:0/C22:6	C_47_H_79_O_13_P	881.518	2005.56
PE	C38:5	C16:0/C22:5	C_43_H_76_O_8_NP	764.523	74.82
	C38:6	C16:0/C22:6	C_43_H_74_O_8_NP	762.5074	211.59
	C44:11	C22:5/C22:6	C_49_H_76_O_8_NP	836.523	20.26
	C44:12	C22:6/C22:6	C_49_H_74_O_8_NP	834.5074	56.60
PS	C28:0	C14:0/C14:0	C_34_H_66_NO_10_P	678.4346	63.49
	C44:12	C22:6/C22:6	C_50_H_74_NO_10_P	878.4972	12.93
PA	C44:12	C22:6/C22:6	C_47_H_69_O_8_P	791.4652	180.21

The two acyl chains of PLs determine the phospholipid diversity. Fatty acids with different structures and characteristics play different roles in the structure and function of PLs. C16:0 and C22:6 were the two major fatty acids of lipids in *S. limacinum* SR21 (Table 4) and also the main fatty acids of acyl chains binding to PLs (Table 3). PC tended to bind to unsaturated fatty acids (UFAs), and the two most important types of PC were PC (16:0/22:6) and PC (22:6/22:6). The proportion of UFAs in PC was 73.3%, of which DHA accounted for 77.7%. Yue [20] et al. also confirmed in *Schizochytrium* sp. A-2 that PC (22:6/22–6) and PC (22:5/22–6) account for half of the total PC. The LPC and PE in this study also showed a preference for binding UFAs. The proportions of UFAs in LPC and PE reached 90.1 and 60.6%, respectively, of which DHA accounted for 80.7% and 78.4%, respectively. PI bound to both SFAs and UFAs, of which DHA accounted for 31.6%. PG and PS tend to bind SFAs, and in this study, they accounted for 75.1 and 83.1% of SFAs, respectively. Eriko [37] found that in *Schizochytrium* sp. F26-b, DHA was the main fatty acid at the acyl ends of PC, LPC, PE, and PI, whereas there was almost no PUFA at the acyl end of PS. Furthermore, Guang [38] also demonstrated that in *S. limacinum*, PUFAs accounted for 86 and 71.6% of PC and PE, and SFA only accounted for 11.8 and 23.5% of PC and PE, respectively. Meanwhile, SFAs in PG reached 64.2%. These results indicate that DHA synthesized by *Schizochytrium* may first be incorporated into PLs, in particular PC, for storage.

#### Phospholipidomic analysis in wild-type and ∆*PLD1* strains

**Fig. 6:**
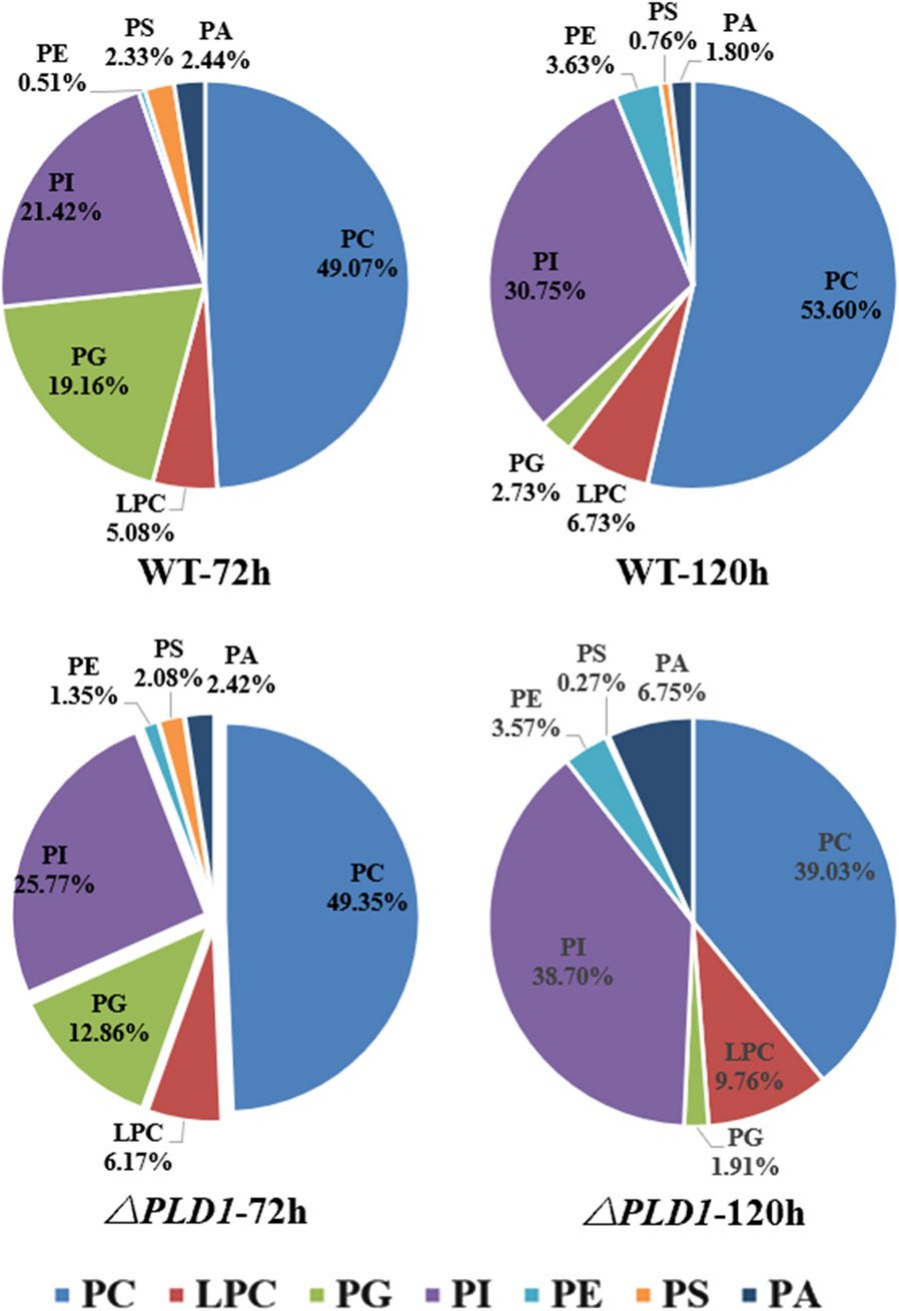
Fractionation of total phospholipids extracted from wild strain and *ΔPLD1* strains at 72 h and 120 h, respectively. *PC* phosphatidylcholine, *LPC* lysophosphatidylcholine, *PG* phosphatidylglycerol, *PI* phosphatidylinositol; *PE* phosphatidylethanolamine; *PS*, phosphatidylserine; *PA*, phospholipid acid. All data were expressed as mean ± standard deviation (mean ± S.D.) of three independent experiments

The proportions of various PLs in the wild-type and ∆*PLD1* strains are shown in Fig. 6. Changes in PL content with fermentation time were compared. The percentage of PG in both strains decreased remarkably as fermentation proceeded, which may be closely related to the cell growth period. The conversion of glucose to glycerol 3-phosphate is much easier compared to that for other phospholipid polar head groups; therefore, it may be easier to synthesize PG with a sufficient carbon source to meet the membrane phospholipid supply during exponential cell production. When glucose is depleted, cells stop proliferating, and membrane PLs metabolism shifts from PL synthesis to interconversion among PLs, resulting in phospholipid composition changes. Furthermore, a decrease in PG levels increases the cellular stress response and lipid dependence of the adaptation mechanism [40]. We observed that the decrease in the proportion of PG was accompanied by a drastic increase in PI. The proportion of PI in the PLs was lower than that of PC. PI has an inositol head group, and its phosphorylation at different positions in inositol is a decisive factor in distinguishing biofilms [41, 42]. PI is also an important signaling molecule that regulates cell signal transduction and the metabolic process [39]. The increase in PI at 120 h in both strains might be the result of a more active signaling pathway after entering the lipid transformation period, especially lipid metabolism, which requires more PI for signal transduction and metabolic regulation. LPC also increased at 120 h in both strains. LPC has one less acyl chain than PC and exhibits a strong surface activity. A high proportion of LPC may cause cell membrane rupture and necrosis [43]. The conversion between PC and LPC is not only related to the acyl remodelling of phospholipids but also to the transfer of fatty acids from PC to TAG, catalyzed by PDAT [44, 45]. The increase in LPC may be due to the rupture and necrosis of cells entering the late phase of growth or might be related to the deacylation of PC to LPC. PC and PA levels showed different changes over time in both strains. In the wild-type strain at 120 h, PC increased by 9.2%, whereas PA decreased by 26.2% compared to the levels at 72 h. In the *PLD1* knockout strain at 120 h, PC decreased by 20.9%, whereas PA increased by 178.9% compared to the levels at 72 h. This further proves that PLD1 mainly plays the role of transesterification rather than hydrolysis of PLs. The knockout of *PLD1* reduced the interconversion among PLs, which affected their composition and strengthened the migration of DHA from PC to TAG at the lipid transformation stage (Fig. 4f). This resulted in decreased PC, increased LPC, and enhanced DHA accumulation.

The changes in the PL content of the different strains were compared. For the three major PLs at 72 h, knockout of *PLD1* resulted in no change in PC, a 32.9% decrease in PG, and a 20.3% increase in PL content compared to the wild-type strain. This suggests that the knockout of *PLD1* mainly reduced PG synthesis at the cell growth stage, which resulted in a decrease in biomass (Fig. 2a). PI not only affects the transport of substances between membranes by regulating ion channels, ion pumps, transporters, endocytosis, and exocytosis but also regulates lipid metabolism and distribution and is closely related to lipid transporters [41]. The increase in PI in the ∆*PLD1* strain demonstrates that the knockout of *PLD1* could upregulate the PI signaling pathway to increase cell metabolism. For other minor PLs at 72 h, knockout of *PLD1* showed no clear change in PA and PS content compared with the wild-type strain; however, LPC and PE content showed a small increase. This increase in PE may be related to the mutual transformation of lipid types in polar lipids. In addition, the phospholipid composition significantly affects the fluidity of the cell membrane, and PE can increase the mobility of the membrane owing to its head group and anionic properties [46]. The increase in LPC was not accompanied by a decrease in PC, indicating that the GPC acylation pathway was activated to synthesize LPC; this is currently thought to be the main pathway for DHA-PC synthesis [47–49]. For PG and PI at 120 h, knockout of *PLD1* resulted in similar changes to those seen at 72 h compared to the wild-type strain. However, PC showed a different change and decreased by 27.2% at 120 h compared to the wild-type strain, indicating that PC had been converted. Accordingly, LPC and PA showed a 45.0 and 275% increase, respectively, at 120 h compared with the wild-type strain, indicating that the acyl migration from PC to DAG to form TAG via the acyl CoA-independent pathway [50, 51] is increased in the ∆*PLD1* strain (Fig. 4f). This requires that more DAG forms TAG via PDAT catalysis, thereby increasing the synthesis of PA to provide more DAG by the Kennedy pathway (Fig. 4c) and resulting in an increase in total lipids (Fig. 2a).

**Fig. 7:**
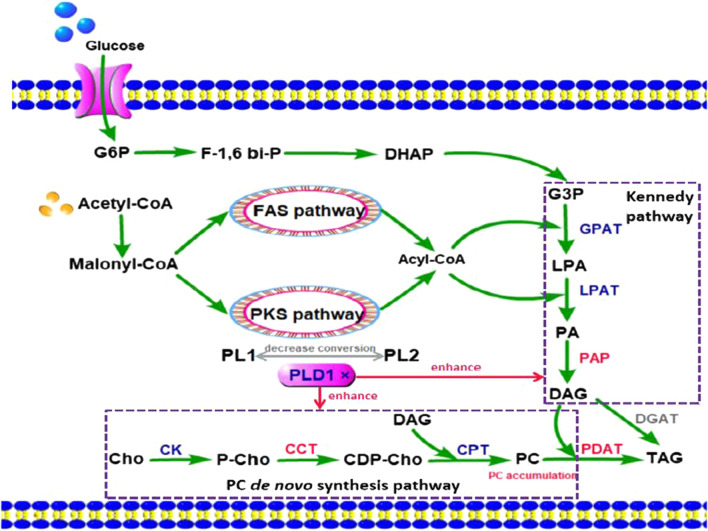
Metabolic mechanism of *PLD1* gene knockout in *S. limacinum* SR21, The red parts indicate that the metabolic pathway or product is enhanced, and the gray parts indicate that the metabolic pathway or product is inhibited

For *S. limacinum* SR21, PC accounted for half of the total PLs (Fig. 6) and tended to bind to UFAs (73.3%) and that DHA accounted for 57.0% of total fatty acids in PC (Table 4). When the transphosphatidyl activity of cells was inhibited by the knockout of PLD1, the compositions of PLs were changed, which might reduce the source of PC from other PLs, thus promoting the de novo synthesis of PC. Therefore, more DHA incorporates into PC to form DHA-PC. The improved *PDAT* expression meant more DHA was migrated from PC to TAG, which resulted in the final increase of DHA production in the ∆*PLD1* strain (Fig. 2d). These results allow us to infer the method by which DHA is synthesized and accumulated in *Schizochytrium*: As shown in Fig. 7, DHA is initially incorporated mainly into PLs, particularly PC, and then migrated to DAG to produce TAG via the acyl CoA-independent pathway depending on PDAT catalysis, which is conducive to the unsaturation of TAGs. It can be promoted by regulating phospholipid metabolism through PLD1.

## Conclusion

This study is the first to explore the effect of *PLD* expression on lipid synthesis in *Schizochytrium*. The genes *PLD1* and *PLD2* identified in *S. limacinum* SR21 have different effects on lipid synthesis. In *S. limacinum* SR21, the knockout of *PLD1* demonstrated a promoting effect on PUFA synthesis and DHA accumulation without affecting biomass and total lipid production. *PLD1* is presumed to play a role in transphosphatidyl activity in *S. limacinum* SR21, and the knockout of *PLD1* reduces PL interconversion and enhances de novo synthesis of PLs, thereby improving the total PL yield and affecting their metabolism. This results in an increase in the binding of DHA to PLs and allows DAG to be assembled into DHA-TAG for storage by an acyl CoA-independent pathway. These results suggest that PLD exerts an important influence on the allocation of fatty acids between TAG and PLs and is closely related to the enzyme activity of PDAT in the acyl CoA-independent pathway. Future studies should investigate its exact mechanisms and functions.

## Materials and methods

### Strains, media, and culture conditions

The *S. limacinum* SR21 (ATCC MYA-1381) strain was purchased from the American Type Culture Collection (Manassas, VA, USA) and was used as the original strain. The seed media and fermentation broths used in this study were the same as those in our previous study [33]. The modified inorganic salt stock solution contained Na_2_SO_4_, 240 g/L; MgSO_4_, 40 g/L; kH_2_PO_4_, 20 g/L; (NH_4_)_2_SO_4_, 20 g/L; K_2_SO_4_, 13 g/L; and KCl, 10 g/L. The medium was sterilized at 115 °C for 20 min before use. The trace element and vitamin solutions were filter-sterilized using a 0.2 μm filter. The activated single colony from plate culture was transferred to the seed medium and cultured for 24 h in the shaker (SKY-2102C double layer constant temperature shaker, Suzhou, China) at 28 °C and 200 rpm. The seed culture (4% v/v) was transferred to the fermentation broth and incubated at 28 °C and 200 rpm for 144 h or longer.

### Mining of *PLD* genes

*PLD*-related information for *S. limacinum* SR21 was obtained from the Joint Genome Institute (JGI) database. The similarity between the *PLD* gene sequence and the corresponding protein sequence was analyzed using DNAstar Megalign (Madison, WI, USA), and phylogenetic tree analysis of the selected *PLD* genes was performed using the NCBI database.

### Construction of gene knockout strains

*PLD* knockout strains were constructed using homologous recombination technology, as shown in Additional file 1: Fig. S5. The up- and downstream sequences of *PLD* were amplified by PCR using primers from *S. limacinum* SR21 genomic DNA (Additional file 1: Table S1). The PCR reagent PrimeSTAR HS (Premix) was purchased from TaKaRa Bio (TaKaRa Biotechnology Co.,Ltd, Dalian, China), and PCR primers were synthesized by Xiamen Boshang Biotechnology Company. The pBlue-zeo plasmid was previously constructed in our laboratory [33] and included an integration region with multiple cloning sites and zeocin expression cassettes for resistance screening. The plasmid pBlue-zeo-*PLD* was constructed by inserting the homologous arm of the *PLD* gene into the multiple cloning site of the vector.

The disrupted fragment was PCR-amplified with primers from the constructed vectors and transformed into *S. limacinum* SR21 by electroporation, according to the method by Ling et al. [33]. The disrupted fragment combined the targeted gene with the homologous arms to replace it [34]. After electroporation, the cells were cultivated for 3 h at 28 °C in a seed medium containing 1 M sorbitol (Macklin Biochemical, Shanghai, China) and then recovered for 3–5 days in a solid medium containing 50 μg/mL zeocin (Sangon Biotech, Shanghai, China). The positive transformants were screened by zeocin resistance plates and cultured at 28 °C and 200 rpm in a shaker (SKY-2102C double-layer thermostatic shaker) for PCR validation and fermentation experiments.

### Measurement of biomass and glucose content

One milliliter of fermentation broth was added to a 1.5 mL centrifuge tube and centrifuged at 10,000 rpm for 2 min at 28 °C. The supernatant was collected to measure glucose concentration using the 3, 5-dinitrosalicylic acid (DNS) method. The cell pellets were washed twice with normal saline and stored in a refrigerator at − 20 °C. The biomass was calculated after 24 h of vacuum freeze drying.

### Lipid extraction and fatty acid composition analysis

Three milliliters of fermentation broth was mixed with 4 mL 12 M HCl and incubated in a water bath at 65 °C for 45 min. Total lipids (TLs) from the mixture were extracted four times with 3 mL of *n*-hexane, and the lipid extract was then purified and dried by evaporation. Total fatty acid (TFA) production was calculated by subtracting unsaponifiable matter (UM) from TLs; UM was isolated from lipids by saponification [52]. The preparation of fatty acid methyl esters and analysis of fatty acid composition were performed as previously described [33].

### Real-time quantitative PCR (qRT-PCR)

Total RNA was extracted from 1 mL of fermentation broth using the RNA Plant Plus reagent (Japan TaKaRa) according to the manufacturer's instructions. The extracted total RNA was reverse transcribed using QuantScript RT kit reagent (Japan TaKaRa) in a PCR machine at 50 °C for 5 min and heated at 85 °C for 5 s to obtain cDNA. Finally, various reaction reagents were added to the PCR octuple using the One Step SYBR PrimeScript PLUS RT-PCR kit (TaKaRa, Japan), and qRT-PCR amplification was performed. Primers used are listed in Additional file 1: Table S2. The mRNA expression level was normalized using the actin gene as the internal control, and the relative gene expression level was calculated using 2^−ΔΔCT^ method [53].

### Quantification of phospholipids

Lipids were first extracted from *S. limacinum* SR21 using the Bligh–Dyer method [54] with some modifications. Five milliliters of fermentation broth was added to 2 mL of deionized water, 3 mL of chloroform, and 6 mL of methanol before ultrasonication for 30 min; 3 mL of chloroform was added for ultrasonication for 30 min. Then, 3 mL of deionized water was added, followed by incubation for 30 min. One milliliter of saturated NaCl solution was then added the solution allowed to stand for 2 h. The solutions were centrifuged at 3000 × *g* for 10 min at 28 °C. After centrifugation, the lower layer of the lipids was transferred to a glass bottle and dried under a nitrogen stream. The obtained lipid samples were weighed and stored at − 20 °C until required. Phospholipids were separated from the lipid samples using solid-phase extraction [55].

Phospholipids were determined using ultra performance liquid chromatography-mass spectrometry (Waters UPLC Acquity H-Class-Xevo-G2 Q-ToF, Milford, MA, USA). The chromatographic experimental conditions were as follows: chromatographic column: ACQUITY UPLC BEH HILIC column (150 × 2.1 mm × 1.7 μm); mobile phase: A is acetonitrile, and B is 20 mM ammonium formate aqueous solution (0.1% formic acid added to obtain pH 3.5); the flow rate was 0.2 mL/min; injection volume: 2 μL; elution procedure: 0–4 min, 95% A and 5% B; 4–22 min, 95–60% A and 5–40% B; 22–25 min, 60% A and 40% B; 25–25.1 min, 60–95% A and 40–5% B; 25.1–30 min, 95% A and 5% B. The mass spectrometry experimental conditions were as follows: electrospray negative ion mode (ESI-); analyzer mode: sensitivity mode; data acquisition time: 2.5–20 min; scanning range: 250–1000 Da; scanning time: 0.5 s; capillary voltage: 2 kV; sample cone voltage: 30 V; extraction cone voltage: 4 V; ion source temperature: 100 °C; desolvation gas temperature: 350 °C; cone gas flow rate: 50 L/h; the desolvation gas flow rate was 400 L/h.

### Statistical analysis

Statistical significance between different strains or groups was evaluated using a *t*-test; 0.01 < *p* < 0.05 was considered statistically significant, and *p* < 0.01 was considered extremely significant. Three parallel experiments were conducted. Values are expressed as the means ± SD (standard deviation).

### Supplementary Information

**Additional file 1:** **Table S1.** Primers for vector construction. **Table S2.** Primers for qRT-PCR. **Fig S1.** Differential analysis of 17 PLD gene (A) and protein sequences (B). **Fig S2.** PLD1 sequences. **Fig S3.** PLD2 sequences. **Fig S4.** Screening of PLD1/PLD2 knockout strains (A, C) and PCR validation (B, D); PC: positive control, NC: negative control, M: marker, PT: knockout strain. **Fig S5.** Schematic diagram of constructing PLD1/PLD2 knockout strains

## Data Availability

The authors can confirm that all relevant data are included in the article and Additional files.

## References

[1] Ren LJ, Sun XM, Ji XJ (2017). Enhancement of docosahexaenoic acid synthesis by manipulation of antioxidant capacity and prevention of oxidative damage in *Schizochytrium sp*. Biores Technol.

[2] Kim TH, Lee K, Oh BR (2021). A novel process for the coproduction of biojet fuel and high-value polyunsaturated fatty acid esters from heterotrophic microalgae *Schizochytrium sp*. ABC101. Renew Energy.

[3] Chi GX, Xu YY, Cao XY (2022). Production of polyunsaturated fatty acids by *Schizochytrium (Aurantiochytrium) sp*. Biotechnol Adv.

[4] Ren LJ, Sun GN, Ji XJ (2014). Compositional shift in lipid fractions during lipid accumulation and turnover in *Schizochytrium sp*. Biores Technol.

[5] Fu J, Chen T, Lu H (2016). Enhancement of docosahexaenoic acid production by low-energy ion implantation coupled with screening method based on Sudan black B staining in *Schizochytrium sp*. Bioresour Technol.

[6] Zhao B, Li Y, Li C (2018). Enhancement of *Schizochytrium* DHA synthesis by plasma mutagenesis aided with malonic acid and zeocin screening. Appl Microbiol Biotechnol.

[7] Metz JG, Roessler P, Facciotti D, Levering C (2001). Production of polyunsaturated fatty acids by polyketide synthases in both prokaryotes and eukaryotes. Science.

[8] Ratledge C (2004). Fatty acid biosynthesis in microorganisms being used for single cell oil production. Biochimie.

[9] Song XJ, Tan YZ, Liu YJ (2013). Different impacts of short-chain fatty acids on saturated and polyunsaturated fatty acid biosynthesis in *Aurantiochytrium sp* SD116. J Agric Food Chem.

[10] Zhao XM, Qiu X (2018). Analysis of the biosynthetic process of fatty acids in *Thraustochytrium*. Biochimie.

[11] Meesapyodsuk D, Qiu X (2016). Biosynthetic mechanism of very long chain polyunsaturated fatty acids in Thraustochytrium sp. 26185. J Lipid Res.

[12] Lippmeier JC, Crawford KS, Owen CB (2009). Characterization of both polyunsaturated fatty acid biosynthetic pathways in *Schizochytrium sp*. Lipids.

[13] Kobayashi T, Sakaguchi K, Matsuda T (2011). Increase of eicosapentaenoic acid in *thraustochytrids* through *thraustochytrid* ubiquitin promoter-driven expression of a fatty acid Δ5 desaturase gene. Appl Environ Microbiol.

[14] Yan JF, Cheng RB, Lin XZ (2013). Overexpression of acetyl-CoA synthetase increased the biomass and fatty acid proportion in *microalga Schizochytrium*. Appl Microbiol Biotechnol.

[15] Li ZP, Meng T, Ling XP (2018). Overexpression of Malonyl-CoA: ACP transacylase in *Schizochytrium sp*. to improve polyunsaturated fatty acid production. J Agric Food Chem.

[16] Wang FZ, Bi YL, Diao JJ (2019). Metabolic engineering to enhance biosynthesis of both docosahexaenoic acid and odd-chain fatty acids in *Schizochytrium sp*. S31. Biotechnol Biofuels.

[17] Metz JG, Kuner J, Rosenzweig B (2009). Biochemical characterization of polyunsaturated fatty acid synthesis in *Schizochytrium*: release of the products as free fatty acids. Plant Physiol Biochem.

[18] Qiu X, Xie X, Meesapyodsuk D (2020). Molecular mechanisms for biosynthesis and assembly of nutritionally important very long chain polyunsaturated fatty acids in microorganism. Prog Lipid Res.

[19] Fan KW, Jiang Y, Faan YW (2007). Lipid characterization of *mangrove thraustochytrid-Schizochytrium mangrovei*. J Agric Food Chem.

[20] Yue XH, Chen WC, Wang ZM (2019). Lipid distribution pattern and transcriptomic insights revealed the potential mechanism of docosahexaenoic acid traffics in *Schizochytrium sp*. A-2. J Agric Food Chem.

[21] Zhao X, Qiu X (2019). Very long chain polyunsaturated fatty acids accumulated in triacylglycerol are channeled from phosphatidylcholine in *Thraustochytrium*. Front Microbiol.

[22] Van Meer G, Voelker DR, Feigenson GW (2008). Membrane lipids: where they are and how they behave. Nat Rev Mol Cell Biol.

[23] Damnjanovic J, Jwasaki Y (2013). Phospholipase D as a catalyst: application in phospholipid synthesis, molecular structure and protein engineering. J Biosci Bioeng.

[24] Yang W, Wang G, Li J (2017). Phospholipase dzeta enhances diacylglycerol flux into Triacylglycerol. Plant Physiol.

[25] Devaiah SP, Pan X, Hong Y (2007). Enhancing seed quality and viability by suppressing phospholipase D in *Arabidopsis*. Plant J.

[26] Lee J, Welti R, Schapaugh WT (2011). Phospholipid and triacylglycerol profiles modified by PLD suppression in soybean seed. Plant Biotechnol J.

[27] Lee J, Welti R, Rotm M (2012). Enhanced seed viability and lipid compositional changes during natural ageing by suppressing phospholipase Dalpha in soybean seed. Plant Biotechnol J.

[28] Bai Y, Jing G, Zhou J (2020). Overexpression of soybean *GmPLD* gamma enhances seed oil content and modulates fatty acid composition in transgenic *Arabidopsis*. Plant Sci.

[29] Zhang G, Bahn SC, Wang G (2019). PLDalpha1-knockdown soybean seeds display higher unsaturated glycerolipid contents and seed vigor in high temperature and humidity environments. Biotechnol Biofuels.

[30] Yaguchi T, Tanaka S, Yokochi T (1997). Production of high yields of docosahexaenoic acid by *Schizochytrium sp*. strain SR21. J Amer Oil Chem Soc.

[31] Weitkunat K, Schumann S, Nickel D (2017). Odd-chain fatty acids as a biomarker for dietary fiber intake: a novel pathway for endogenous production from propionate. Am J Clin Nutr.

[32] Pfeuffer M, Jaudszus A (2016). Pentadecanoic and heptadecanoic acids: multifaceted odd-chain fatty zcids. Adv Nutr.

[33] Ling X, Zhou H, Yang Q (2020). Functions of enyolreductase (ER) domains of PKS cluster in lipid synthesis and enhancement of PUFAs accumulation in *Schizochytrium limacinum* SR21 using triclosan as a regulator of ER. Microorganisms.

[34] Li ZP, Meng T, Ling XP (2018). Overexpression of malonyl-CoA: ACP transacylase in *Schizochytrium sp*. to improve polyunsaturated fatty acid production. J Agric Food Chem.

[35] Gong YM, Wan X, Jiang ML (2014). Metabolic engineering of microorganisms to produce omega-3 very long-chain polyunsaturated fatty acids. Prog Lipid Res.

[36] Li ZP, Chen X, Li J (2018). Functions of PKS genes in lipid synthesis of *Schizochytrium sp*. by gene disruption and metabolomics analysis. Marine Biotechnol.

[37] Abe E, Hayashi Y, Hama Y (2006). A novel phosphatidylcholine which contains pentadecanoic acid at sn-1 and docosahexaenoic acid at sn-2 in *Schizochytrium sp*. F26-b. J Biochem.

[38] Wang G, Wang T (2012). Characterization of lipid components in two microalgae for biofuel application. J Am Oil Chem Soc.

[39] Cantley LC (2002). The phosphoinositide 3-kinase pathway. Science.

[40] Rowlett VW, Mallampalli V, Karlstaedt A (2017). Impact of membrane phospholipid alterations in *Escherichia coli* on cellular function and bacterial stress adaptation. J Bacteriol.

[41] Balla T (2013). Phosphoinositides: tiny lipids with giant impact on cell regulation. Physiol Rev.

[42] Kutateladze TG (2010). Translation of the phosphoinositide code by PI effectors. Nat Chem Biol.

[43] Moog R, Brandl M, Schubert R (2000). Effect of nucleoside analogues and oligonucleotides on hydrolysis of liposomal phospholipids. Int J Pharm.

[44] Bates PD, Ohlrogge JB, Pollard M (2007). Incorporation of newly synthesized fatty acids into cytosolic glycerolipids in pea leaves occurs via acyl editing. J Biol Chem.

[45] Bates PD, Durrett TP, Ohlrogge JB (2009). Analysis of acyl fluxes through multiple pathways of triacylglycerol synthesis in developing soybean embryos. Plant Physiol.

[46] Hu XC, Luo YY, Man YL (2022). Lipidomic and transcriptomic analysis reveals the self-regulation mechanism of *Schizochytrium sp*. in response to temperature stresses. Algal Res.

[47] Kjell S, Andrea CN, Hans R (2008). Identification of a novel GPCAT activity and a new pathway for phosphatidylcholine biosyhthesis in *S. cerevisiae*. J Lipid Res.

[48] Eriko A, Kazutaka I, Eri N (2014). Novel lysopholipid acyltransferase PLAT1 of *Aurantiochytrium limacinum* F26-b responsible for generation of palmitate-docosahexaenoate-phosphatidylcholine and phosphatidylethanolamine. PLoS ONE.

[49] Will RK, Jana PV (2020). Role of the *Candida albicans* glycerophosphocholine acyltransferase, Gpc1, in phosphatidylcholine biosynthesis and cell physiology. FASEB J.

[50] Dahlqvist A, Stahl U, Lenman M (2000). Phospholipid:diacylglycero acyltransferase: an enzyme that catalyzes the acyl-CoA-independent formation of triacylglycerol in yeast and plants. P Natl Acad Sci USA.

[51] Bates PD, Browse J (2011). The pathway of triacylglycerol synthesis through phosphatidylcholine in *Arabidopsis* produces a bottleneck for the accumulation of unusual fatty acids in transgenic seeds. Plant J.

[52] Dhara R, Bhattacharyya DK, Ghosh M (2010). Analysis of sterol and other components present in unsaponifiable matters of mahua, sal and mango kernel oil. J Oleo Sci.

[53] Larionov A, Krause A, Miller W (2005). A standard curve based method for relative real time PCR data processing. BMC Bioinform.

[54] Bligh EG, Dyer WJ (1959). Extraction of lipids in solution by the method of bligh & dyer. Can J Biochem Physiol.

[55] Donato P, Cacciola F, Cichello F (2011). Determination of phospholipids in milk samples by means of hydrophilic interaction liquid chromatography coupled to evaporative light scattering and mass spectrometry detection. J Chromatogr A.

