# Supplementary material for: Knockout of a PLD gene in Schizochytrium limacinum SR21 enhances docosahexaenoic acid accumulation by modulation of the phospholipid profile
Source: Biotechnol Biofuels Bioprod. 2024 Jan 30;17:16. doi: 10.1186/s13068-024-02465-w (PMC10826259; doi:10.1186/s13068-024-02465-w)
Supplement: Supplementary file 1 — Additional file 1: Table S1. Primers for vector construction. Table S2. Primers for qRT-PCR. Fig S1. Differential analysis of 17 PLD gene (A) and protein sequences (B). Fig S2. PLD1 sequences. Fig S3. PLD2 sequences. Fig S4. Screening of PLD1/PLD2 knockout strains (A, C) and PCR validation (B, D); PC: positive control, NC: negative control, M: marker, PT: knockout strain. Fig S5. Schematic diagram of constructing PLD1/PLD2 knockout strains [file 13068_2024_2465_MOESM1_ESM.pdf]

Table S1 Primers for vector construction

Primers	Sequence（5’→3’）
PLD1-U-F	CCGCTCGAGACGCACGTGATGAATTCCA
PLD1-U-R	CCCAAGCTTTGTTCTGCTTCCACTCATT
PLD1-D-F	CGGGATCCTTGCGTCTCCCATGCTA
PLD1-D-R	GCTCTAGAGCTGCTGCCGAGGCTTA
PLD2-U-F	CCGCTCGAGGGCCGGTGAAAGTCATAA
PLD2-U-R	CCCAAGCTTGGGCAAGCATTGTGCAAG
PLD2-D-F	CGGGATCCTAGAGTAATGCATTATTTTCTT
PLD2-D-R	GCTCTAGAACTTGAAAATACCATTAGGTAG
AN0-F	TCTCAAAGATTAAGGCATGCATGTGT
AN0-R	TTCACCTTCCTCTAAACAATAAGATT

Table S2 Primers for qRT-PCR

Primers	Sequence（5’→3’）
PLD1-RT-F	ATTGGGGTTCCATTGCTCGT
PLD1-RT-R	TTTTCAAACTCGCGCAGGTG
PLD2-RT-F	CCAGAGCCGGAGTTTTCAGT
PLD2-RT-R	TGCTCCACTCCCCCATATCA
PAP-RT-F	CAAATCCAGCAACAACCAAAACAAC
PAP-RT-R	GCAGTAGGGTCCTCAGAATAATGAA
FAS-RT-F	TGAGGCGTTTGCCAACTACA
FAS-RT-R	GCTCCTTGACAATGACCATG
CLF-RT-F	TCCGTTCTGCCTAAGGAA
CLF-RT-R	GAGAGCCGTCTTGATGAG
CCT-RT-F	CAGCTTTCACGGACCCTCTT
CCT-RT-R	GCCAGCGTGAAGTCAAAGTG
CDS-RT-F	TCTACGAATACCACGCGCTC
CDS-RT-R	GAAGAATCCACCAAACGGCG

A

Percent Identity (upper triangle) / Divergence (lower triangle)

	1	2	3	4	5	6	7	8	9	10	11	12	13	14	15	16	17		
1		19.0	20.6	19.4	19.4	19.9	20.3	19.6	20.2	19.8	19.4	20.2	21.5	20.0	20.3	19.5	20.6	1	PLD-100463.seq
2	197.5		20.1	22.2	23.4	23.0	22.8	22.2	22.4	21.6	20.9	22.1	20.0	21.5	21.4	20.1	21.8	2	PLD-115221.seq
3	216.5	117.7		21.8	21.3	20.8	20.9	22.3	21.3	19.7	21.4	19.0	20.5	21.0	21.4	21.1	19.3	3	PLD-116877.seq
4	323.6	109.9	99.4		19.6	20.8	21.0	21.0	20.6	21.2	20.5	19.4	19.8	19.4	22.1	20.8	19.3	4	PLD-119909.seq
5	197.6	55.0	130.5	124.1		21.2	20.1	20.3	20.9	20.0	20.9	18.9	20.2	20.2	21.2	20.1	20.7	5	PLD-136168.seq
6	203.1	83.4	133.7	128.3	74.2		19.3	20.1	19.2	19.2	21.4	20.3	19.9	20.4	21.6	18.1	20.1	6	PLD-140350.seq
7	203.5	161.7	164.0	207.8	175.6	182.4		21.6	21.6	22.2	21.4	21.1	19.9	20.7	21.5	22.6	21.7	7	PLD-143421.seq
8	221.7	120.8	56.6	96.4	115.3	130.1	207.3		22.0	20.2	22.3	19.9	19.3	20.3	22.7	21.4	19.9	8	PLD-146704.seq
9	164.2	142.3	100.7	116.4	119.8	137.5	183.9	107.6		21.0	23.2	20.7	22.1	19.4	22.2	19.0	19.5	9	PLD-2990.seq
10	260.7	191.8	197.5	197.3	202.5	187.0	101.8	197.4	196.5		20.4	20.3	19.6	20.0	20.7	22.5	22.1	10	PLD-30965.seq
11	199.5	121.4	103.9	104.2	129.6	137.4	178.6	98.6	74.6	210.8		20.2	19.8	18.9	23.2	20.8	20.3	11	PLD-3794.seq
12	213.9	195.2	172.5	165.0	192.9	167.6	106.6	171.9	182.7	115.7	174.8		19.5	19.4	20.7	22.2	20.1	12	PLD-39595.seq
13	57.5	158.6	179.5	179.1	172.7	219.7	168.8	170.1	159.5	299.2	190.1	188.1		20.5	19.8	20.3	20.8	13	PLD-46.seq
14	66.8	144.1	165.8	180.9	181.6	153.3	177.1	174.7	187.8	197.9	166.9	255.4	57.1		19.6	21.2	19.9	14	PLD-6429.seq
15	164.4	109.1	113.1	106.9	124.2	143.7	173.1	119.9	84.3	183.1	58.5	167.0	154.5	139.0		20.8	20.6	15	PLD-8088.seq
16	186.1	210.7	189.1	211.8	258.9	158.2	65.8	233.3	176.8	105.2	182.0	107.7	234.9	185.6	153.9		21.1	16	PLD-9259.seq
17	211.1	308.2	350.0	200.5	246.6	227.8	132.8	241.4	233.0	116.2	222.2	111.6	265.9	192.7	350.0	145.5		17	PLD-9317.seq
	1	2	3	4	5	6	7	8	9	10	11	12	13	14	15	16	17		

B

Percent Identity (upper triangle) / Divergence (lower triangle)

	1	2	3	4	5	6	7	8	9	10	11	12	13	14	15	16	17		
1		9.8	10.0	10.2	10.2	10.5	10.2	12.1	10.5	9.8	9.8	11.3	13.7	9.4	10.5	9.9	10.4	1	PLD-100463.pro
2	1000.0		13.3	13.9	14.9	15.7	12.9	13.7	13.9	13.1	13.5	15.1	11.9	12.9	12.9	11.8	12.9	2	PLD-115221.pro
3	626.0	736.0		11.3	12.2	12.0	11.8	13.4	12.1	10.7	12.9	11.3	11.9	10.9	12.1	12.6	9.5	3	PLD-116877.pro
4	907.0	285.0	1000.0		12.5	13.8	12.9	12.6	12.1	12.7	12.4	11.8	11.0	12.1	13.0	14.4	12.0	4	PLD-119909.pro
5	569.0	493.0	511.0	371.0		12.2	12.6	11.1	11.9	11.3	12.6	11.1	11.6	10.9	11.8	13.0	12.8	5	PLD-136168.pro
6	649.0	261.0	487.0	313.0	442.0		10.9	12.2	12.3	12.0	13.3	11.2	11.5	10.9	11.4	13.0	11.2	6	PLD-140350.pro
7	1000.0	1000.0	615.0	1000.0	615.0	845.0		12.6	10.2	13.2	12.4	12.7	10.9	11.7	12.5	14.6	13.2	7	PLD-143421.pro
8	615.0	482.0	291.0	545.0	466.0	466.0	531.0		13.4	12.7	13.6	11.1	10.8	11.0	13.4	13.6	11.3	8	PLD-146704.pro
9	1000.0	754.0	360.0	577.0	569.0	754.0	775.0	396.0		12.5	14.5	13.1	12.4	10.2	12.5	10.3	10.1	9	PLD-2990.pro
10	1000.0	1000.0	907.0	1000.0	1000.0	626.0	335.0	545.0	531.0		12.5	12.3	10.5	11.2	12.3	12.8	13.2	10	PLD-30964.pro
11	1000.0	374.0	321.0	504.0	560.0	482.0	661.0	303.0	274.0	1000.0		11.5	11.0	11.3	15.2	12.8	12.9	11	PLD-3794.pro
12	1000.0	945.0	595.0	736.0	1000.0	1000.0	389.0	504.0	545.0	301.0	637.0		10.8	11.2	12.3	13.4	12.8	12	PLD-39595.pro
13	173.2	1000.0	796.0	688.0	1000.0	661.0	845.0	649.0	703.0	1000.0	553.0	874.0		11.8	12.1	11.3	10.9	13	PLD-46.pro
14	1000.0	399.0	451.0	688.0	661.0	426.0	466.0	426.0	461.0	545.0	371.0	504.0	569.0		11.8	12.0	10.5	14	PLD-6429.pro
15	1000.0	447.0	476.0	524.0	719.0	577.0	1000.0	418.0	289.0	688.0	241.0	736.0	796.0	430.0		13.0	11.4	15	PLD-8088.pro
16	1000.0	775.0	1000.0	538.0	626.0	553.0	299.0	517.0	504.0	447.0	1000.0	471.0	595.0	410.0	703.0		15.1	16	PLD-9259.pro
17	1000.0	626.0	1000.0	524.0	615.0	1000.0	303.0	545.0	615.0	380.0	688.0	403.0	1000.0	451.0	1000.0	224.0		17	PLD-9317.pro
	1	2	3	4	5	6	7	8	9	10	11	12	13	14	15	16	17		

Fig. S1 Differential analysis of 17 PLD gene (A) and protein sequences (B)

ATGAGCAATGGGAGAGATGACAAAAGGGGGTCTGAGCCGCTTGGCTGCGG
CCACGAGGGCCACTTTACCATGCTTGCGTCCAAGACGCCCGGTGTTCCTCC
TGAGGACGACTTTGGTCTTGGCATAGTCGATACCGCTGTTGCTCGCGAAGG
CAGCCACAGTCGTCATCGTACTGCATCAACCTCAGGACCAACATTATCAAC
ATCATCAACAACAACTCCAGCTCAAGCTTCAGGCCAAAGAAATTCTCAAG
CCAACAATGAATCGAAGCGACAGAGCAAATTATTGAAAGATCCAGAGGGA
GATGCAAAGCCCAACGACGAGGAGAAAGAAGATCAACAGCCGTCGCATG
GCGACAAGATTTCACCGGTATTAAAATCTCAAACCTCGAACCCGCAAGAGC
GACACCCTAAACACAAAAAGACGCGTCGCCAAAGAAGTAGAACAAGCAG
CAAGGGAACTCTAGCAGCCAAACAAAATAGTACAGGAACCTCGGCAGCAC
AACCACCTAGACCACGACTCTTAACTGGAAATCGATCCTCGTCGGTATTTA
CTATTATGATGATGATGACACAAGGTCTCGTCAATCAAGTACTATCGGCCTG
GGATTTGTACCAGAGGTCCCCAATGTAATGCGAAGGGATAGCGGCGTAACA
TTGGACATGTTTGTAATTCCAGAGCCGGAGTTTTCAGTAAATGAAACCATC
ACCAACGAGCAATCTACCTTAAATATTCCGAATCTCCAAGTTGGAGGAGAA
ATCGTCTACAGAATCTGGCTAAATTTTGGTGATATGGGGGAGTGGAGCATTT
ACGTCACTCGTACGGACCTCTCTGCTCTGATCTCTAAACTTAAACTTAATCG
TATGCTCGCTATCAAAACAAACATCACCAATATTGGCCAGAGACTTGGCCG
TCACAAATCCATGGATCCGGGGCGCCCTCAGTTAGAACGCTCACCTAACTC
GAGGCGAATTATCTTGCCTCAAGTTTCAGAGCGTAACGTTCTTCAAGATCA
AGCTATGAACCATCATCATCATCAGCAGCAGCATCGAGATCAGAACCAGAA
TAAACAACGTGGTCGCTCTCCTCAACGGGCTACCTCACACCATGGTGGCGA
TCAGCAAGATTCGTCATCCCAGCACTCAACTGGACCAGACGGCCATCTTGT
TTTGCGAATGAGTAAATTATTGTTTGTGCCCGCCATGGGTTCGGCGGCGCGC
ATTCGGAAGGTCAAAGCTGATCGTCGTCGATTGGTTAAGTCTATCTTTCAAT
CTATTTTGTGCAACGAAACGATCCGTAATGCGCCATTTGTGAAAGAATTCCT
CTGCATTAGTACATTGACCTTTGACACACGATTCGGAAAGACATATAAAGA
AGGCTGGGTCCGGGTTACGGTGACGAAGCGAGATCGTGAGAACGAGAAGT
TACAATGGAAGGGGAAAATTTCTTGTTTCTGTTGTATTTGCGCTTGTGCGCC
AGACCCGCAACGTGCTAAGACATTTTTGCAACCAAGCTATCGCCGGAACCG
CTGGCTATGGGCAGTAGTTAAGCCATCATTTATTGCATTCTTTGATCAGAGC
CCTCATGAACTTGACGCACGTGAGGCAAGTTTCGTCATTTTGTATTCCCACA
ACTATGAGATCAATAAAAGCTACTCCACAACTGGCTCACACCGTGCAATTC
ACGTTGTTGGAGATTCTTTGAATGTTCGCCTCAAACTGCCAACTAGATGGC
AAAAGAAGGTGTGGTCACGTGCTATGCGCGACGCCTTTCAAGCTTCCTCTG
TTCTTGCTAACGCTGAAGGGAATGATCAAGTCGCAGATGCGGGTGTGTCTG
CTGGCTGGTGTGACACGCACCAAAATCAATCCTTTGCGCCACGGCGACTCC
TTCGTGACGAGCCAGGTGCTGAGATGTGTATGGCTAAATTCCATATTGATGC
TGAAAATTATTTAAAGCGGTGTACGAGGCTATTGACAGCGCAGAGAGCCA
AATCTTTATCCAAGGATGGTGGGTCTGCGCGAACTTGCCATTACTGCGCCCC
TCCCGAAGTAACGACAAAGAAAACACCCTTGGTGGTATCCTAGAGAAGAA
GGCAAAGCAAGGTGTGAAAATTTCTATTCTCATGTACAATGAGGTATCCCTT
GCCCTTCCTTTAGATAGTGCATTCCAAGAAAAAGAGTTTCGTAAACTTGAG
GGTCAGCAGAACATCTCTGTTTTACGCCATCCAATGCACAAGATTAAGAAA

AACTCAATCTGGTTTTGGAGTCATCATGAGAAAATTGTGGTCATTGATCAG
ACGCATTCGTTCTGTGGAGGCATTGACTTATCCTGGGGACGTTATGACACA
CATTCACATCGATTGTACGATCCACATGCACATGCACGTTCTGATGTTGGGC
GAGAATGGCCTGGTATCGACTTTTCTAACGTTCGTGATCATGATTTCGAGAA
TGTCTCGGACTTCTCACGCATTCAGCACGCTGGGGATTGTCCACGAATGCC
TTGGCATGATGTTCAATGTGAATATTGGGGTTCCATTGCTCGTGATATCGCTC
GGCACTTTATCCATCAATGGAACCACGCTCGTTTTGCTAATGCTGATGAAAC
CCTGGCTGATGCCCTTGTGCCTATGGATGTACTACCTCATCAGTACCGCGTT
AACTTTGCTGAGATTGCTGCTGGTCAACCTGTCACAATTGCCCCTCACCTG
CGCGAGTTTGAAAACGATGATGATAATCTGAGTGATGAAGGCCCTGCTGAA
GATATCGGCCCCCCAGGTAGCCAGCCATCTTCTCCAGTGTCGTCCTTGCCA
AGAAGTCGCTCGAATTCACCTGTTCCTCGTTGGTCTCAGGCCAAAGATGAC
GACATTCAGTCCTTGCCAACTATTACTGAAAGTGATATCGATGAAGAGGAG
CAGATTGGTAGAGAAGATGATGATGACAGTGATGGCTCTCTTCCGCCCCCT
CCAGAGCTACACGGAGAGATGATCACAGAGCCAACAGCTGTCCCCCCACC
AAATGTTACAATGAATGGAGAAGCTGCTCATCAGAACGGTTTCCACAACAA
AAAGAAGTCTGGACCTCAAGCTCCTAATCCAGAGCTAGATATGGATGGGAA
ATCGCAGGAGAGTAGGCCTGCTTCCATGTCGGTTTGCAGCTGTGCTACACA
AGGTATTAATGAGAACGTCGTTTACCACGTTAACGCGCAAGTTGTTCGAAG
TCTCGGGCTTTGGTCAGGTTCTACACCTGGCGAGAAAGAGCGATCAATTGA
GAAAGCAATGCGCAATCTCATTGAAGACGCAGAGCGTTTTATTTATATTGAG
AATCAGTTCTTTATCTCCGGCTTGGACTCTGATAATGAGGTTGCAAATCGTG
TTCTGGATGCTCTTTTCAAACGTATCCTTCGTGCGCATCAAGCCAGGGCTGA
GTTCAAGGTGATGATTTTAATTCCATTAATTCCTGGATTTGATGGATATATCT
CCGAATCGCCATCCATTCGCCGTGTGCTCTTTTATGAGTATCGATGTATCAAT
CGGGGAAGCCAATCCTTGTTCTACAACCTCTTGCGAAAAGGAATCAATCCA
GATGACTATGTCCACTTTTTTGGGCTCCGTGCCTGGGAACCGCTTTCGTTTG
GAAGCTCAAAGGCAGCAGAGGTACTTGGCATGCGTCGAGTTGCAACTGAG
CTAGTATATGTCCACTCCAAGGTCCTGGTAGTAGACGATGTTAAATGCATAA
TCGGCAGTGGGAATATAAATGATCGTAGTCTAACAGGAAACCGTGACACCG
AGTTGGCCGTAGTCATTGAGGCATCTAATACGGGAAAGAACTTTGTTCGAA
ACTTTCGTCTCTCTCTCATGTCTGAACACTGGGGTATTCAAGATAATCCACA
TGACCCGTTATGGAAACAGCTTCGTACACCATGGCACAAAGAAAATTTTAA
GGAGCTCTGTAATATCTCTCAAACAAACACGCAAATATTCGAAGACATATTT
CCATGCATTCCCCGCGATGATGTGCGCGATTCAACAGACCTCCGTAACTTCT
CGCGTGAGCGTTACACCGTGATAGAATCTTCGAATGCGATTGAGGATGAGC
TAAGTCACATCAAAGGTCACATCGTGGACTTTCCTCTGCGATTTCTGGAAC
AATACGACCTTAAACCAGGTCTCATTGAAGATACTTCTGGCGCACTTTTGG
GCAACGAGTTATTCATCTGA

Fig. S2 *PLD1* sequences

ATGCAATGTTTACCGAACTGCTGCAAGTGTGGACTCTTTGGTCTTTCGGATC
CATTTGTCAAAGTAAAGCTTGACAATGCGAAGATTTGCCGATCGACTGTGT
GCAACAATACCTTACGTCCTAATTGGAATGAGTATCTGAGCATGGATGTTGC
TCACGATGCAAAGACTATTGTCTTCCGGGTTATGGATGAAGATAGTCTTGGC
GATCAGTACCTTGGCCAGGTTGCCTTCTCCTGGACCCAGATTCTTCAAAAT
GGCAAAACGCTGGAGGGCGAATTTCCGATCTCGCGCATAAGTATGGTTGGC
TCACAATTAAGATGCGCTACACCCCGCACCAACATGAACCCTCATCGTCTC
ACCAACATTCATCCATATTCTGTACCGAACACGTATTTCCCACCTCGCCGTG
GTGATCATTTTGTGCTCTACCAAAGTGCTCACCAACCTGGTATTGGTAATGT
GTTGCCTCAAATTGAGCTAGGTGAGGAGGGCGAAGATCTTGAACCCTTTCG
CGCCCGTCCTTGCTGGAAGGATCTCTATAATGCGATCGATGCCGCAGAAAA
CTTCATTTGTATTTGTGGATGGTCTGTAAACCCTTTCATTCGTTTGGTGCGG
ACAGGCCCTGATCACAAGAACAAATTGACCCTTGGAAATCTTTTGAAGAA
GAAGGCCAAGGAAGGTGTTATCGTCCTTATCATGGTATGGGATGACCTTTCT
AGTACATCCATGACCTCCGGTTTGATGGGAACCATGGACGAAGAATGTGTC
AAATTCTTCGAGGACTCACCTGTTGTTGCAGTCAAAACACCTCGTGTAGAC
CAACGCGGTCTTATCAGTAGTTTGGCAAACCTCTCGTTTGCATATACTCATC
ATCAGAAGTCTATTATCATGGATGTTAAACCTGAGAACGCAGCTCGCCTTCC
TATTACCGCTTTCTTAGGAGGTATTGATGTAACCTCAGGTCGATACGATGATT
CAGATAAACCTTTGTTTGCTTCTCTACACCGCACTCACAGAGATGATTACTA
TCAAAATTGTCTACCTGCAGAGGTTGCTGATCCAGAGAAAGGTCCGCGCCA
GCCATGGCAAGATATTCACACCCGCGTTACTGGGCGAGCGGCAGTGGATGT
ACTCAAAAACTTCATTGAGCGCTGGCACATGCAAGCAGGCAACCATCGGC
ATCCATTAAGTGGATGCACGGGAAGCCGGGCTAAGTGGATTGCCCAAACTC
GTGAGAGGGCGGGTTCCTGGAATGAGGGAGTGTTTGCTCAATCCAATAAC
GATAGGGTAGTGCAGGTGGTACGGTCCATTAATGAAGACTCCGCACGTTTA
GAAAAGTCTATCCATGCCCCTAACGCGTTTGCCGACAAGGGTATCAAAGTA
GATAACAGTATTCATAAGGCCTATGTGCACCACATCCGGTCTGCTAAACACT
TCATCTACATCGAATCGCAGTATTTTATTGGTGGATGCCATATGTGGAGCAA
ATCCAAAAGAAGTGGAGCAAGCAATCTGATTCCGATTGAACTAGCTTCCAA
GGTGTGCAGTAAGATTGCTGCCAACGAAGCCTTCCACGTTTACCTAGTAAT
CCCTCTCTTCTGCGAAGGTCTGCCAGCAGACAAGGCAGTGATGCAAATTCT
TCGGTATCAGTATTTCACAGTCGCGATGGTATACCGTCGTATTCATGAAGCA
CTTAAAAAACACAAGCTAAAGGATCGCTCCGTGACTGACTACTTTTCGGTC
TTTTTCTTGGGTAACCGTGAAGCTGAAGAGACCACCCGACTGGAAGCCGA
GCAGGGGCGCGAATTTTGCGAAGAGGATTATGAGCAGGTTGCTCATGCCGC
ACAAAAACGTCGTCGCTTCCAAATTTACGTACACTCCAAGCTCCTCATTGT
TGATGATGCCGTCGCCGTTATTGGCAGTGCAAACATTAACCAACGGTCTTTT
GATGGTTCGAGAGATACAGAAATTGGAGTTTCGATGTACGAGATGGCTCGA
GTCGCGACGGAAAAATCTACGCCGCGTGGCCTCATTTATGGATTTCGGATG
TCCCTTTGGGCAGAGCATCTTGGAGGCTCTATTACTGATGACGAGGACTTT
GATCCTTCGATTCTACAGCGTCCCTCATCCGTCGAGTGCGTCCGTCTGATTC
AGACACGGGCTTATAAGAACTGGCAGTACTACGTTGATGATGAGCACAACG
AAAATGTCCCTGGACATTTGATGACATACCCATACAAGGTTGACCACGACA

CTGGTAAAGTGACTACCTTGGACAATCTCGTTCACTTTCCCGATTTCCCTCA
TTCACGTATCCTCGGAGCGAAAACTAAACTTCCAGATGAACTCACAACTTA
A

Fig. S3 *PLD2* sequences

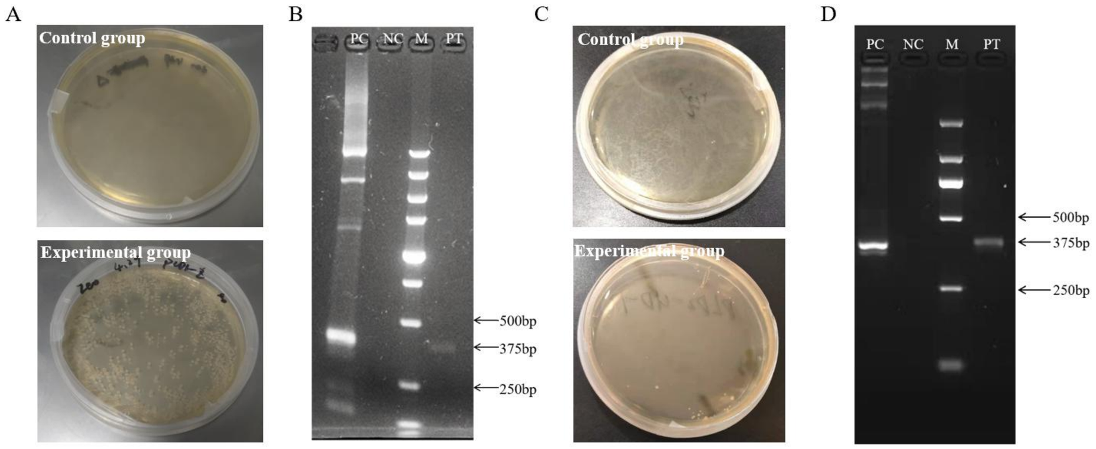

Fig. S4 Screening of *PLD1*/*PLD2* knockout strains (A, C) and PCR validation (B, D); PC: positive control, NC: negative control, M: marker, PT: knockout strain

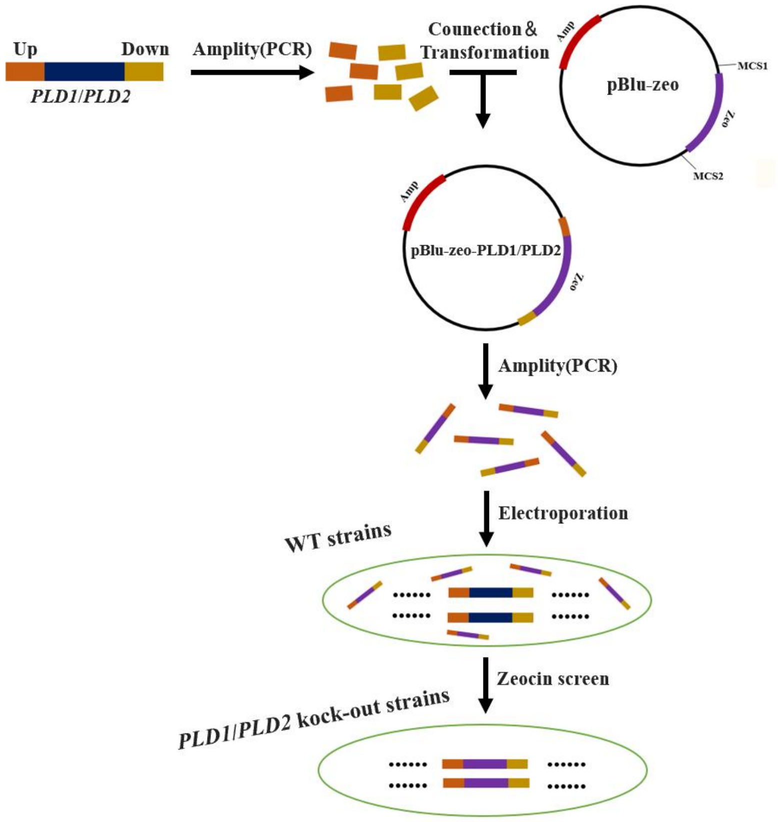

Fig. S5 Schematic diagram of constructing *PLD1/PLD2* knockout strains
